# Mobility-based real-time economic monitoring amid the COVID-19 pandemic

**DOI:** 10.1038/s41598-021-92134-x

**Published:** 2021-06-22

**Authors:** Alessandro Spelta, Paolo Pagnottoni

**Affiliations:** Department of Economics and Management, University of Pavia, 27100 Pavia, Italy

**Keywords:** Statistical physics, thermodynamics and nonlinear dynamics, Complex networks, Nonlinear phenomena

## Abstract

Mobility restrictions have been identified as key non-pharmaceutical interventions to limit the spread of the SARS-COV-2 epidemics. However, these interventions present significant drawbacks to the social fabric and negative outcomes for the real economy. In this paper we propose a real-time monitoring framework for tracking the economic consequences of various forms of mobility reductions involving European countries. We adopt a granular representation of mobility patterns during both the first and second waves of SARS-COV-2 in Italy, Germany, France and Spain to provide an analytical characterization of the rate of losses of industrial production by means of a nowcasting methodology. Our approach exploits the information encoded in massive datasets of human mobility provided by Facebook and Google, which are published at higher frequencies than the target economic variables, in order to obtain an early estimate before the official data becomes available. Our results show, in first place, the ability of mobility-related policies to induce a contraction of mobility patterns across jurisdictions. Besides this contraction, we observe a substitution effect which increases mobility within jurisdictions. Secondly, we show how industrial production strictly follows the dynamics of population commuting patterns and of human mobility trends, which thus provide information on the day-by-day variations in countries’ economic activities. Our work, besides shedding light on how policy interventions targeted to induce a mobility contraction impact the real economy, constitutes a practical toolbox for helping governments to design appropriate and balanced policy actions aimed at containing the SARS-COV-2 spread, while mitigating the detrimental effect on the economy. Our study reveals how complex mobility patterns can have unequal consequences to economic losses across countries and call for a more tailored implementation of restrictions to balance the containment of contagion with the need to sustain economic activities.

## Introduction

 The novel coronavirus disease (COVID-19), caused by the Severe Acute Respiratory Syndrome Coronavirus 2 (SARS-CoV-2), has hit the globe in a massive way. Although the coronavirus family encompasses the virus which led to the Severe Acute Respiratory Syndrome (SARS) in 2003, the rapid surge in positive individuals and the early evidence of greater transmission indicated that SARS-CoV-2 was more contagious than previous coronaviruses and, therefore, potentially more dangerous for humans^1,2^. As a matter of fact, despite the huge efforts put into place by worldwide governments to limit the spread of the epidemics, the world counts approximately 152 million reported positive cases and 3.1 million total fatalities as of 1 May 2021.

From a socio-economic viewpoint, SARS-COV-2 has forced many governments around the world to implement several non-pharmaceutical interventions (NPIs)^3–5^. In the epidemiological context, NPIs refer to any methods which contrast the spread of an epidemic disease without the use of pharmaceutical drug treatments. They consist of a set of measures that can be employed at any time, and are used in the period between the emergence of an epidemic disease and the deployment of an effective vaccine. The US Centers for Disease Control and Prevention (CDC) distinguishes across “Personal NPIs” (hand washing, cough and sneeze covering, quarantine), “Community NPIs” (social distancing and closures) and “Environmental NPIs” (routine surface cleaning). Amongst the various forms of NPIs, mobility restrictions have been playing a key role in reducing SARS-CoV-2 transmission, with lockdowns exerting the most substantial impacts^4,6^.

As a consequence, considerable effort has been recently put in quantifying the effects of human mobility on the spread of SARS-CoV-2. Recent groundwork shows how mobility habits are significant explanatory variables for the number of newly reported COVID-19 infections^7^, and concludes that SARS-CoV-2 spread is more a matter of network interconnectivity rather than of spatial proximity^8^. As the infection rate needs to be cut down drastically and rapidly to observe a consistent decrease of the epidemic spread and mortality rate^9^, government policies aimed at contrasting SARS-CoV-2 diffusion have been sudden and numerous, with direct impact on human mobility, which in turn facilitates the emergence of trend breaks in the number of reported SARS-CoV-2 cases and significantly reduces the virus transmission^10,11^.

Policy restrictions have been identified as key elements to effectively limit the spread of the virus^12–17^, thus prompting the adoption of lockdown interventions based on mobility restrictions as key strategies to limit the contagion. On the other hand, such policy interventions have been recognized to induce severe disruptions on mobility patterns^13,18^ and to determine relevant economic consequences as a large portion of disposable workers, i.e. individuals not infected and available to work, is prevented from keeping up working activities^19–22^. For this reason, a big effort is put in understanding the appropriate balance between the direct effect of mobility restrictions on the spreading of contagion and the indirect consequences caused on economic outcomes^23–25^. For instance, there is a flourishing literature employing mobility networks to study the diffusion of SARS-COV2^3–5,12,14,15,26,27^. These works combine an epidemiological perspective with a complex system approach, able to capture non-linear relationships in the dynamics of the underlying system, with the aim of understanding the impact of lockdown measures on the virus transmission. Moreover, recent studies have recognized how mobility reductions induce harsh negative consequences on economic systems during the lockdown phase, producing both a loss of aggregate economic output^22,28–31^ and a contraction of consumption expenditures^32–34^. The economic assessment of mobility restriction measures is indeed of great interest for policy makers and motivates a growing literature related to the investigation and measurement of trade-offs between the need to limit the spread of contagion and the provision of adequate levels of economic output^24,25,35,36^. In fact, evidence has shown that mobility restrictions condition both the shape of mobility networks and the body of economic systems, by affecting the inter-dependencies among geographical zones^29,37–39^.

Monitoring the economic performance over time is a fundamental aspect of economic analysis and a key requirement for policymakers. One of the most commonly known and used indicators in this context is the Industrial Production Index. Industrial production is a measure of output of the industrial sector of the economy, with the industrial sector includes sub-sectors such as mining, manufacturing, electricity, gas and steam and air-conditioning. The Industrial Production Index specifically measures changes in the production volume of an economy (or country), hence providing a measure which is free of price change influences, making it a suitable indicator of economic activity. The index is measured against a reference period (in our case, the year 2015 is taken as a reference) and expresses the change in the volume of production output with respect to such baseline value. In other words, the Industrial Production Index monitors temporal changes in the value added of an economy’s industrial sector, thus boasting a close relationship with the performance of the economy as a whole. Although the Organisation for Economic Co-operation and Development (OECD) provides production indicators for the aggregate industry and for the manufacturing and construction industries, our focus is on the total production index, as it measures the total output of the industrial sector of a country’s economy, and is therefore able to monitor a country’s economic activity in its entirety. Our focus is on industrial production rather than GDP, as the latter measures the aggregate final value of goods and services produced, which comprise those that can actually be provided under smart working regimes.

Against this background, we exploit the relationship existing between the different dimensions of human mobility and the real economic activity of a country, so to provide a timely monitoring indicator of the state of the economy. In other words, we investigate the interplay between the SARS-COV-2 NPIs, such as mobility restrictions, and the effects caused by such restrictions on the productive system of four representative European countries. In particular, this study proposes an explicit characterization of the industrial production dynamics which accounts for mobility patterns in Italy, France, Germany and Spain; the EU member states counting the largest number of total SARS-CoV-2 confirmed cases as of February 2021, as well as the top four countries in terms of industrial production over recent years. Taking into account for the heterogeneous characteristics of commuting patterns, we assess the economic consequences of various forms of lockdown policies, by studying the impact of human mobility patterns on the aggregate industrial production through a dynamic factor model^40^ (see Methods).

Our real-time monitoring approach is grounded on the nowcasting methodology. Nowcasting stands for the prediction of the present, the very near future, and the very recent past states of variables^41^. The term stems from meteorology as a contraction of the terms “now” and “forecasting”, where it indicates the practice of weather forecasting on a very short term mesoscale period. Originally based on heuristic rules, it now relies on sophisticated statistical and econometric models. The nowcasting methodology has recently become appealing to economists to assess the current state of a country’s economic activity, through synthetic measures such as its gross domestic product (GDP) or industrial production, which are available with a significant delay, a low frequency and might be subject to revisions^42–47^. Central banks, such as the US Federal Reserve and the European Central Bank, make use of nowcasting tools to monitor the state of the economy on a real-time basis, generating high-frequency estimates of the official statistics (see Methods).

The basic principle of nowcasting is the exploitation of information which is published at higher frequencies than a target variable of interest to obtain an early estimate of such variable before the official figure becomes available. In other words, within the nowcasting framework, by exploiting information on mobility patterns, we are able to build a dynamic process which provides day-by-day estimates of industrial production statistics that are announced at a monthly frequency and with long delays, thus providing policymakers a valuable tool to evaluate the magnitude of the effects of mobility restrictions on the real economy.

We rely on two unique human mobility datasets provided by Facebook (FB) and Google (GOOG) with the aim of building a model able to track the day-by-day economic activity of a country. Through its Data for Good program^48^, Facebook provides near real-time massive datasets describing the phone-tracking based movements of individuals within and across country’s administrative regions. To help researchers in understanding the pandemic, Google has also released its Community Mobility Reports, tracking changes in mobility of people via mobile phones across location categories, among which retail and recreation, grocery stores and pharmacies, workplaces and transit stations (see Methods).

The contribution of this paper is twofold. Firstly, we provide a comprehensive analysis of mobility patterns both from a geographical viewpoint, i.e. mobility flows across administrative regions, and from a categorical perspective, meaning the categories of places from/to which individuals flow. At this investigation stage, we study how these two dimensions of human mobility are related to the implementation of government policy restrictions, as measured by the Oxford COVID-19 Government Response Tracker (OxCGRT), which collects information on the type and severity of policy actions undertaken by governments (see Methods). Secondly, we exploit mobility data to assess the day-by-day changes on the countries’ economic output, as measured by their industrial production. Given that economic variables are generally low-frequency ones (e.g. industrial production is released monthly) and published with a certain delay, our proposal is of utmost importance with regards to policy makers’ decision making processes. Indeed, we offer an extensive tool to timely monitor and analyze the impact of mobility restrictions on a country’s economic activity. By combining our real-time economic activity tracker together with epidemiological models, policy makers are able to evaluate the trade-off between economic and health damages due to the implementation of restrictive measures. In other words, they can monitor both the epidemic and economic side-effects of their restrictive measures, through the impact they exert on human mobility.

**Figure 1:**
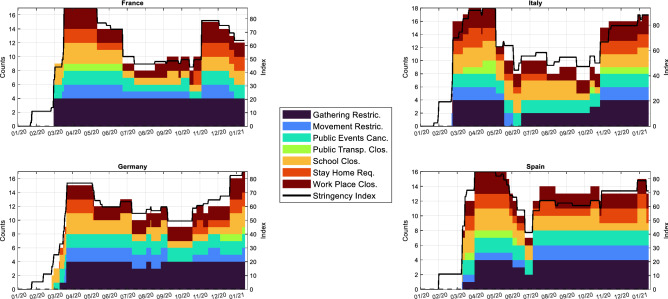
Line diagrams and bar-charts showing Government policy responses. The figure reports the line diagrams and bar-charts expressing the evolution of government policy responses in France, Italy, Germany and Spain over the starting year of the pandemic. Colored bars indicate the number of measures put in place within each category. Solid black lines indicate the Oxford Stringency Index (OxCGRT), an additive score of nine indicators recording information on containment and closure policies, such as school closures and restrictions in movement, and health policies. The left vertical axes represent the number of policies (counts) put in place by the Italian, Spanish, French and German governments, while the right vertical axes report the Oxford Stringency Index. The horizontal axes represent time (data on government policies are available at a daily frequency).

In Europe, on March 9th, 2020 the Italian Prime Minister announced the extension of the stringent lockdown measure previously adopted for 26 northern Italian provinces to the whole peninsula, with restrictions on mobility to that necessary for work and family emergencies, coming into effect the next day. In the wake of the Italian government, on March 14th the Spanish government formally declared state of emergency over coronavirus, issuing an order of general confinement for more than 46 million people. On March 17th, within the French country a strict nationwide lockdown came into force, with measures imposing stay-at-home, except for grocery shopping and other essential tasks. Unlike other European countries, Germany had stopped short of ordering stringent restrictions to its over 80 million population in the first instance, opting for strict social distancing measures which were issued on March 22nd.

Since the imposition of the first hard restrictive measures, governmental policy responses to limit the spread of the disease have been of various nature (see Fig. 1). Most countries started in March with restricting gatherings, closing schools and cancelling public events, soon extending their interventions to stay-at-home requirements, workplace closures and movement restrictions. After a first phase of stringent lockdown, governments acted with a gradual relaxation of the harshest restrictive measures until the summer period, when many restrictions, especially those concerning mobility and stay-at-home requirements, were lifted. The relaxation was, however, not uniform across countries: the German government has maintained a higher level of alert in terms of the number and of stringency of policies adopted, along with the Spain, if compared to Italy and France. With the winter and the second wave around the corner, governments resumed a large portion of mobility restrictions and stay-at-home requirements, besides exacerbating those already in place. Government policy actions have shocked human mobility patterns in a substantial way, with direct impacts on both SARS-CoV-2 spread and economic activity.

## Results

**Figure 2:**
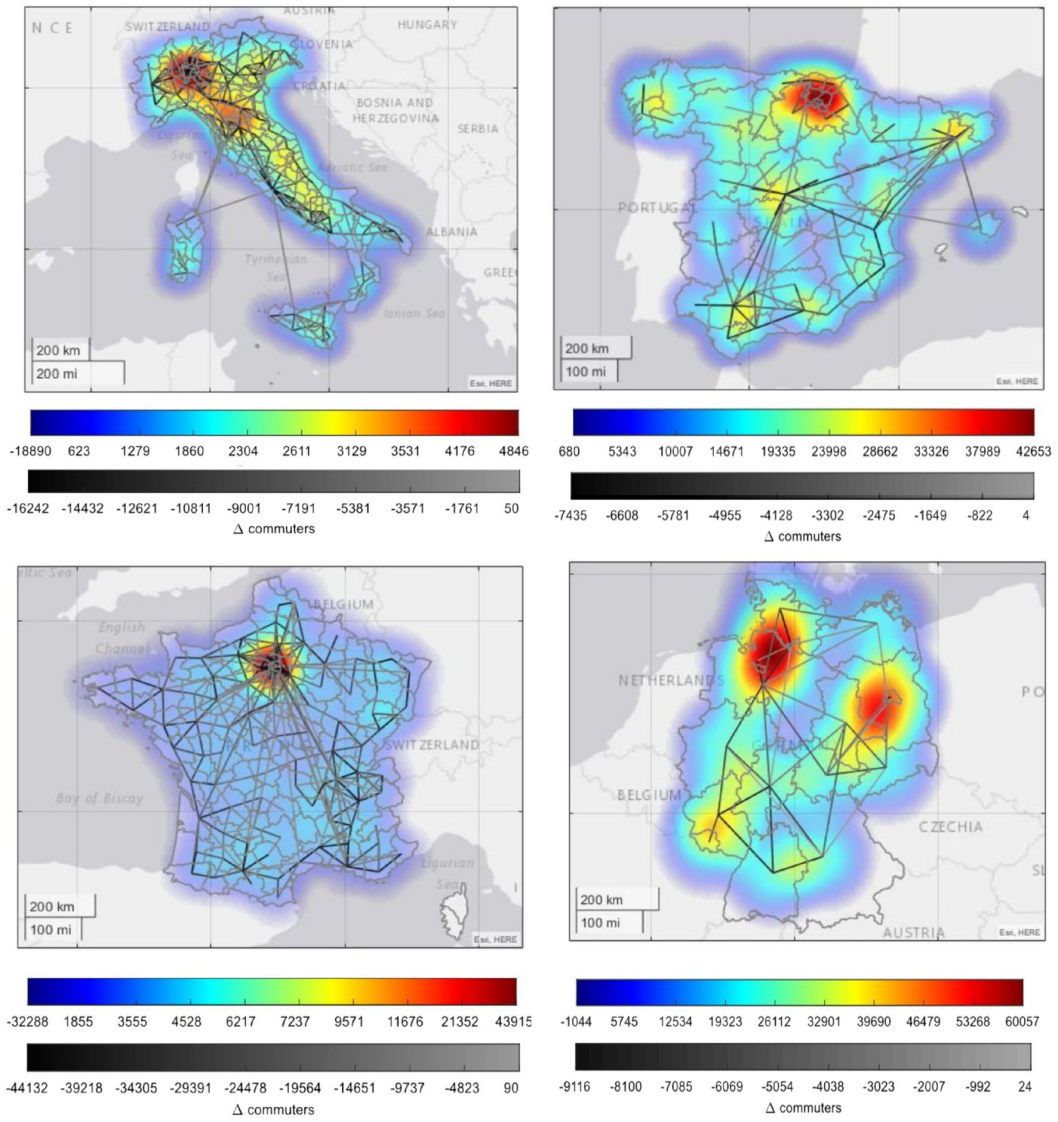
Heatmaps and line diagrams of the geography of commuting patterns. The figure reports the heatmaps and the line diagrams representing the mobile-phone-based commuting difference ($\Delta$ *commuters*) in Italy, Spain, France and Germany at relevant dates within the first wave of the pandemic for each country with respect to baseline values (pre-pandemic period). In particular, the figure shows the difference in the number of moving people measured at relevant dates (first day of national lockdown) with respect to a baseline value (computed as the average of the three weeks prior to the lockdown). The color map indicates the difference in the number of commuting people within administrative regions, and the grey-intensity links indicate the difference in the amount of individuals flowing between administrative polygons. Administrative regions are at NUTS3 level (province) for Italy, Spain and France, while for Germany data are available at NUTS2 level (regional). Relevant dates represent the first day of lockdown for Italy (10 March 2020), Spain (15 March 2020), France (17 March 2020) and the first date available for Germany (25 March 2020).

The nationwide lockdown measures imposed with the beginning of the epidemic spread exerted a dramatic impact on different dimensions of human mobility. Figure 2 shows the geographic distribution of the difference in commuting patterns with respect to the pre-pandemic phase, within and between countries’ administrative regions at the points in time when the first lockdown measures where imposed, while Supplementary Fig. S1 in SI illustrates the dynamics of commuting flows during a baseline period. The figure highlights a substitution effect concerning the mobility between and within jurisdictions. Indeed, we observe a striking decrease in the commuting flows between administrative regions, accompanied by a surge in the commuting flows within them. The difference in the mobility between geographic zones comes as a natural consequence of the fact that restrictive measures did not allow people to move outside their own administrative regions. On the other hand, evidence suggests that people who were banned to travel outside their administrative regions tended to move more around their neighborhood, thus increasing the degree of mobility within their region.

In general, the largest absolute variations in mobility are registered in the areas in which metropolitan cities are located, where there naturally exists a high degree of mobility both within and between the geographic zones during normal business periods. This mostly involves the northern part of Italy, with Milan and Emilia-Romagna, which count industrial districts spread over the entire areas, the north-central part of Spain, the metropolitan area of Paris in France and the industrial districts of Köln and Düsseldorf in Germany, along with part of the eastern regions. However, there are some exceptions: the metropolitan areas of Madrid and Barcelona, along with Bavaria and the western regions of Germany, register a noticeable difference in the mobility from/to outside the regions, while they do not show such a large impact on the mobility within the area. This is arguably because many outside residents have left their living areas to go back to their places of origin.

**Figure 3:**
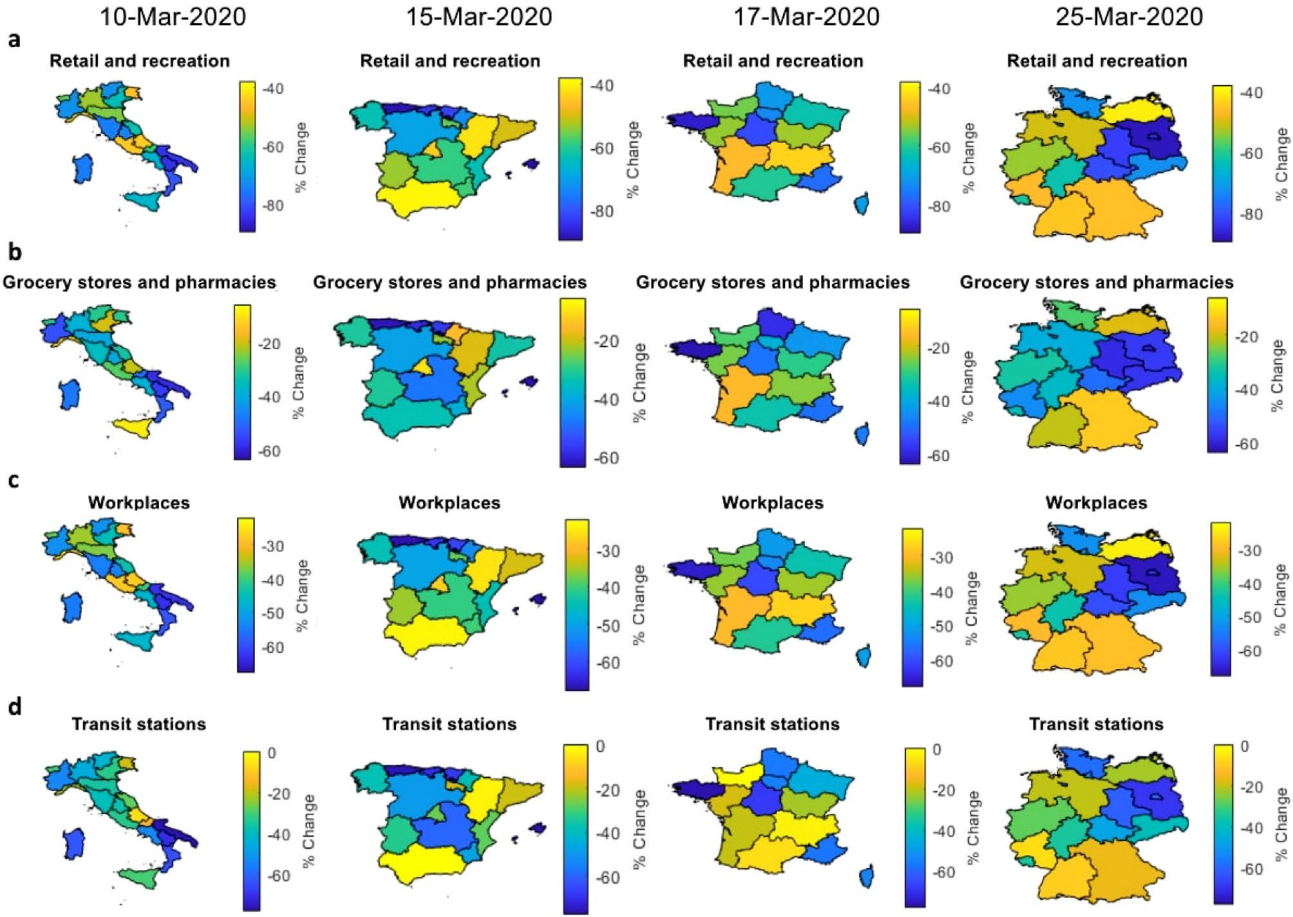
Heatmaps expressing the geography of mobility trends. The figure reports as heatmaps the phone-tracking-based changes in mobility patterns of individuals to retail and recreation (**a**), grocery stores and pharmacies (**b**), workplaces (**c**) and transit stations (**d**) across administrative regions in Italy, Spain, France and Germany at relevant dates within the first wave of the pandemic. Colors indicate the differences in percentage terms with respect to baseline. Administrative regions are at NUTS2 level (regional) for all the countries under analysis. Relevant dates represent the first day of lockdown for Italy (10 March 2020), Spain (15 March 2020), France (17 March 2020) and the first date available for Germany (25 March 2020).

The impact of lockdowns is not only tied to the magnitude of commuting flows, but also to the mobility trends across different categories of locations. Figure 3 illustrates the geographic distribution of changes in human mobility trends for retail and recreation (Fig. 3a), groceries and pharmacies (Fig. 3b), workplaces (Fig. 3c) and transit station (Fig. 3d) in the countries’ administrative regions before and after the first lockdown measures. People’s movements to retail and recreation sites were the ones most hardly hit by the imposition of lockdown measures, followed by those to transit stations and workplaces. Given the non-essentiality and consequent closure of retail and recreation places, all non-food product groups had shown extremely steep drops in their retail trade volumes, in particular the decline for textiles, clothes and footwear was drastic. Stores selling “essentials” such as groceries and pharmacies, on the contrary, did witness a gentler decrease in the number of visitors. On top of that, a few regions in Italy, especially the northern ones - firstly hit by the spread of the virus -, even exhibited increases in the mobility to groceries and pharmacies, which is traceable to the panicked shoppers stocking up on supplies and clearing out many supermarkets’ shelves amid the surge of SARS-CoV-2.

Changes in people’s habits are highly heterogeneous across countries and regions. Spain is the country showing the largest falls in people’s mobility across different categories: northern regions, with the exception of Aragon, are those suffering from the highest drop in mobility across types of locations, with movements to retail and recreation dropping up to 90%. The magnitude of mobility variations across different location categories of France and Germany are quite comparable. In Germany, southern regions tend to be less affected by changes in mobility, whereas in eastern Germany such changes are generally steeper. Movement across categories in France are quite heterogeneous; however, north western regions appear to be the mostly affected ones, and Brittany specifically. Italy saw generally more moderate decreases, with the steeper downturns of mobility in a cluster of southern regions consisting of Apulia, Campania, Basilicata and Calabria, arguably because they were not among the northern areas which had already been quarantined before the nationwide lockdown which, in turn, showed lower drops in the relative mobility across location categories.

Generally speaking, the effects of policy interventions and virus spread have been determinant to the evolution of human mobility flows over time, and exerted heterogeneous effects both across countries and geographic zones, and over time (see Supplementary Fig. S2 in S1). The distribution of the distance travelled by commuters is generally centered around short path ranges during the first lockdown phases, while it gradually flattens by displaying heavy tails, along with the various lifts of mobility restrictions. With the advent of the second wave after summer, the distribution becomes gradually less dispersed, indicating that people tend to move less, behaving more homogeneously. However, by comparing the timing of two waves of infections and the associated mobility restriction policies, in the latter wave we observe a more heterogeneity in people’s behavior, meaning that mobility restrictions did not reach the same effectiveness they achieved during the first nationwide lockdown phases, except for Germany. Further, the distribution of the number of commuters shows a general heterogeneity of the quantity of people moving within and between administrative regions during the months which follow the periods of strict mobility restrictions, during which a portion of people started moving more than they did during lockdowns, while others kept their behavior constant regardless of the severity of the measures imposed.

**Figure 4:**
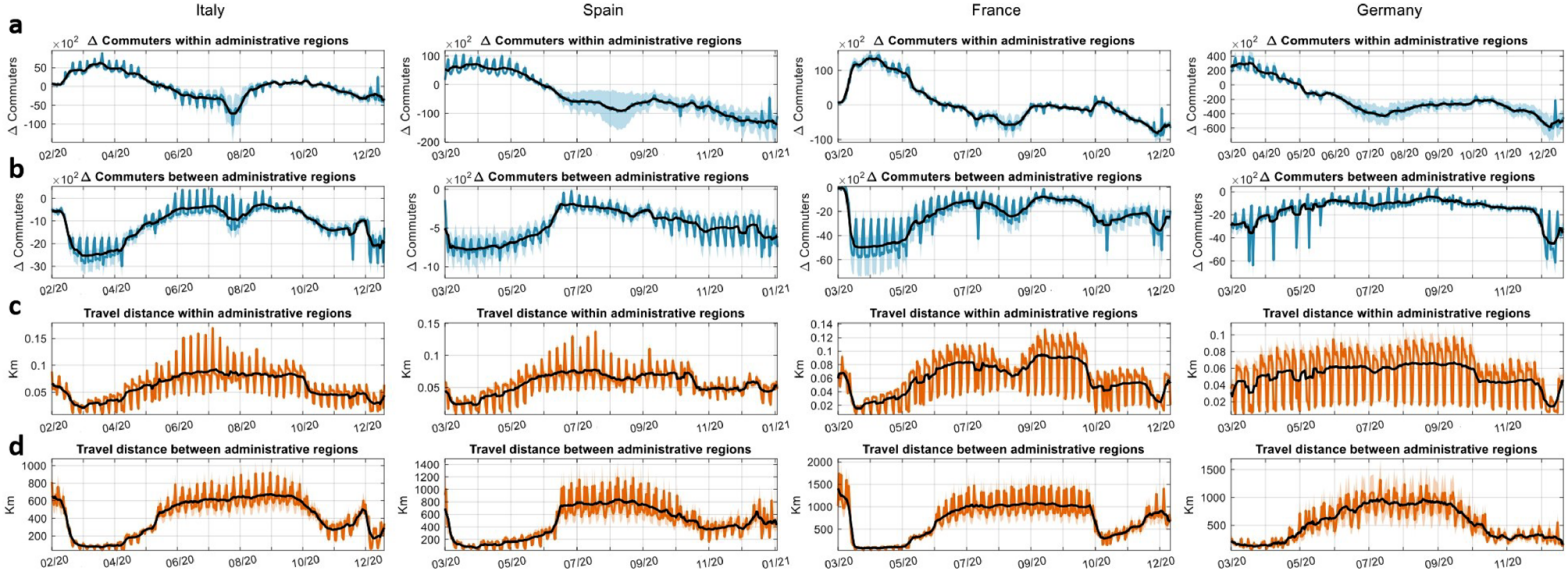
Line diagrams of the commuting patterns dynamics. The line diagram show the evolution of the mobile-phone-based commuting difference ($\Delta$ *Commuters*) within (**a**) and between (**b**) administrative regions with respect to baseline and the average travel distance (km) within (**c**) and between (**d**) administrative regions in Italy, Spain, France and Germany. In particular, the mobile-phone-based commuting difference is computed with respect to a baseline value (the average of the three weeks prior to the lockdowns). Colored solid lines represent the real average time series dynamics, black solid lines show the 7-day moving averages, shaded areas indicate the 1-standard deviation bands. Administrative regions are at NUTS3 level (province) for Italy, Spain and France, while for Germany data are available at NUTS2 level (regional).

Additionally, in order to have insights on the evolution of mobility patterns in time, Fig. 4 depicts the dynamics of the number of people’s commuting over time (Fig. 4a,b) and their travelled distance (Fig. 4c,d), averaged across administrative regions. As the raw commuting time series exhibit weekend and holiday-related effects, we analyze the 7-day moving average (of both the commuting within administrative regions $c^{W}_{av}$ and between jurisdictions $c^{B}_{av}$ and the relative within travelled distance $d^{W}_{av}$ and between jurisdictions $d^{B}_{av}$) to better identify their overall trend, as well as the respective standard deviation ($c^{W}_{std}$, $c^{B}_{std}$, $d^{W}_{std}$ and $d^{B}_{std}$), which highlight differences in mobility behaviors across administrative regions. These quantities, together with their relative Google counterparts, will be used as explanatory variables for assessing the impact of commuting patterns and mobility trends on industrial production. We observe a significant reduction of mobility flows between provinces during the whole pandemic development, which does rarely touch its pre-crisis levels. The substitution effect involving commuting flows within and between provinces is particularly evident during the first nationwide lockdown phases, whereas the effect is offset during the summer period, when many restriction measures were lifted, giving raise to a significant variability in the mobility across administrative regions. After that, the new season of policies brought a different effect with respect to the ones adopted during the first wave: a gentler decrease in the number of commuters between provinces, but this time together with a drop in the number of commuters within provinces. Reading this together with the distance travelled, the first wave policies seem to have impacted more strongly people’s mobility than the second ones, although not without some exceptions - see the effects of Germany’s Christmas lockdown amid the SARS-CoV-2 surge on overall mobility.

**Figure 5:**
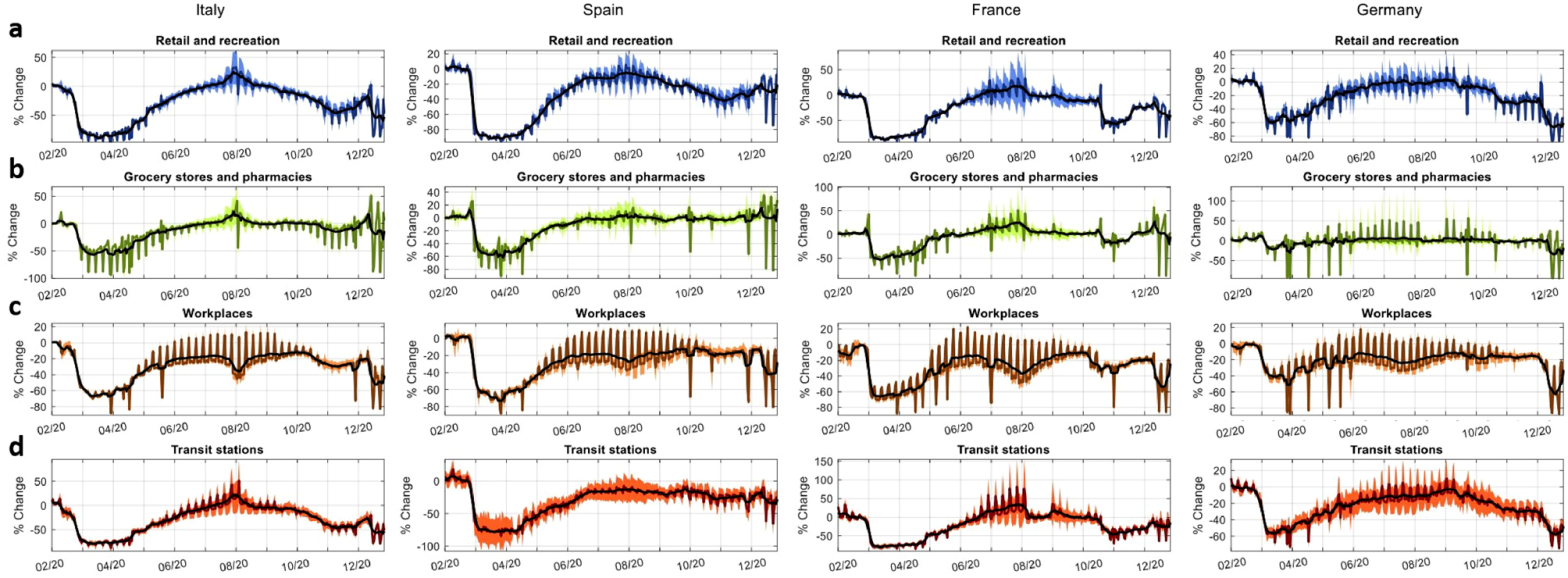
Line diagrams of the Google mobility trends dynamics. The line diagrams show the average dynamic of phone-tracking-based changes in mobility patterns of individuals to retail and recreation (**a**), grocery stores and pharmacies (**b**), workplaces (**c**) and transit stations (**d**) across administrative regions in Italy, Spain, France and Germany. Colored solid lines represent the real average time series dynamics, black solid lines show the 7-day moving averages, shaded areas indicate the 1-standard deviation bands. Administrative regions are at NUTS2 level (regional) for all the countries under analysis.

The sequence of policy restrictions and lockdown lifting have also radically changed people’s behavior over time. Figure 5 shows the dynamics of human mobility trends for retail and recreation ($r_{av}$ and $r_{std}$), groceries and pharmacies ($g_{av}$ and $g_{std}$), workplaces ($w_{av}$ and $w_{std}$) and transit station ($s_{av}$ and $s_{std}$) in the countries’ administrative regions. The figure reports an overall slowdown in mobility during the first hard lockdown phase, which exhibits variations of even more than 90% with respect to baseline in some cases - see, e.g.  movements to retail and recreation sites (Fig. 5a). Given their essential nature, grocery stores and pharmacies (see Fig. 5b) were the least affected in terms of number of visitors, although the policy interventions and virus spread pushed people to stock up and reach them less frequently than usual, registering drops of around 50% across countries - except for Germany. The summer period, when many restriction measures were lifted, shows a reversion of mobility trends. Retail and recreation (Fig. 5a) and transit stations (Fig. 5d) were the categories mostly interested by a recovery of previous mobility patterns, in most cases turning to values in the neighborhood of normal business periods. Not only people tended to move more (e.g. for leisure and vacations), but they also spent more time in shopping and recreation activities, giving origin to the so-called “revenge spending” phenomenon, for which wealthy individuals emerging from isolation overcompensate by splurging more than they routinely spent before the pandemic. Consistently with the dynamic of commuting flows, mobility trends highlight that people’s response to policies of the second wave were weaker than those linked to the first hard lockdown. The drop in mobility across categories of interest is in most cases much gentler, with the only exception of Germany, which is undoubtedly the country suffering the most from the restrictive measures adopted concomitantly with the second wave.

Policy interventions, through the channels of closures and mobility restrictions, exerted an astonishing impact on the countries’ economic outputs, which show highly correlated patterns over the pandemic year - see Supplementary Fig. S3, together with Supplementary Tables T1, T2 in SI. The first large contractions in the monthly industrial production ($Y^M$) were registered in March 2020, when the Italian industrial production dropped by approximately 28%, followed by the French (17%), the Spanish (13%) and the German (11%) ones. The sharp economic downturn continued in the month of April, with all countries witnessing an industrial production decline between 20 and 21%, and Italy bearing the harshest consequences of containment policies. From May onwards, $Y^M$ exhibits an extraordinary recovery, bouncing back - despite not homogeneously - nearby their pre-crisis levels by the summer period. Industrial production series show then a gradual slowdown in recovery, and start again slightly decreasing in November, right after the rise in policy interventions to contrast the second wave, except for Germany. Indeed, the German recovery was slower but sustainable over time, in contrast to the faster but fleeting Italian one, which led to a significant drop of the industrial production already in September. The dynamics of the industrial production is strictly linked to the human mobility figures in each country (see Supplementary Fig. S4 in SI). As a matter of fact, industrial production shows large positive correlations both with commuting patterns and mobility trends, while it shows a negative correlation of comparable magnitude with the mobility within administrative regions, due to the aforementioned substitution effect in commuting patterns between to within administrative polygons. This result holds for all countries analyzed.

Mobility data constitute the basis for the day-by-day estimation of the evolution of the industrial production. Within this perspective, by leveraging information contained in mobility changes, our aim is to produce a daily nowcast of the change in the industrial production rather then to the variable in level. To obtain such estimates, we first take the first difference of all time series so to ensure the stationarity property of our series is met (see Supplementary Table T3 in SI). Secondly, we need to specify the factor loading structure. The factor loading resolves into a single global factor ($f^G$), which affects all variables, and few additional local blocks, which control for idiosyncrasies in particular subgroups of series. This choice improves inference, ensuring the model is robust to the presence of local correlations. In particular, to model local correlations, we include four additional blocks ($f^{AV_{FB}}$, $f^{STD_{FB}}$, $f^{AV_{GOOG}}$ and $f^{STD_{GOOG}}$). In particular, these blocks refer to the average Facebook mobility values ($f^{AV_{FB}}$), their standard deviation ($f^{STD_{FB}}$), the average Google mobility values ($f^{AV_{GOOG}}$) and standard deviation ($f^{STD_{GOOG}}$). The specified model is run and updated daily starting from the end of September 2020, while parameters are re-estimated at the beginning of each month. The daily updates quantify how each new change in mobility patterns contributes to updates in the industrial production nowcasts, while common factors varies either if mobility changes or the model parameters are re-estimated.

**Figure 6:**
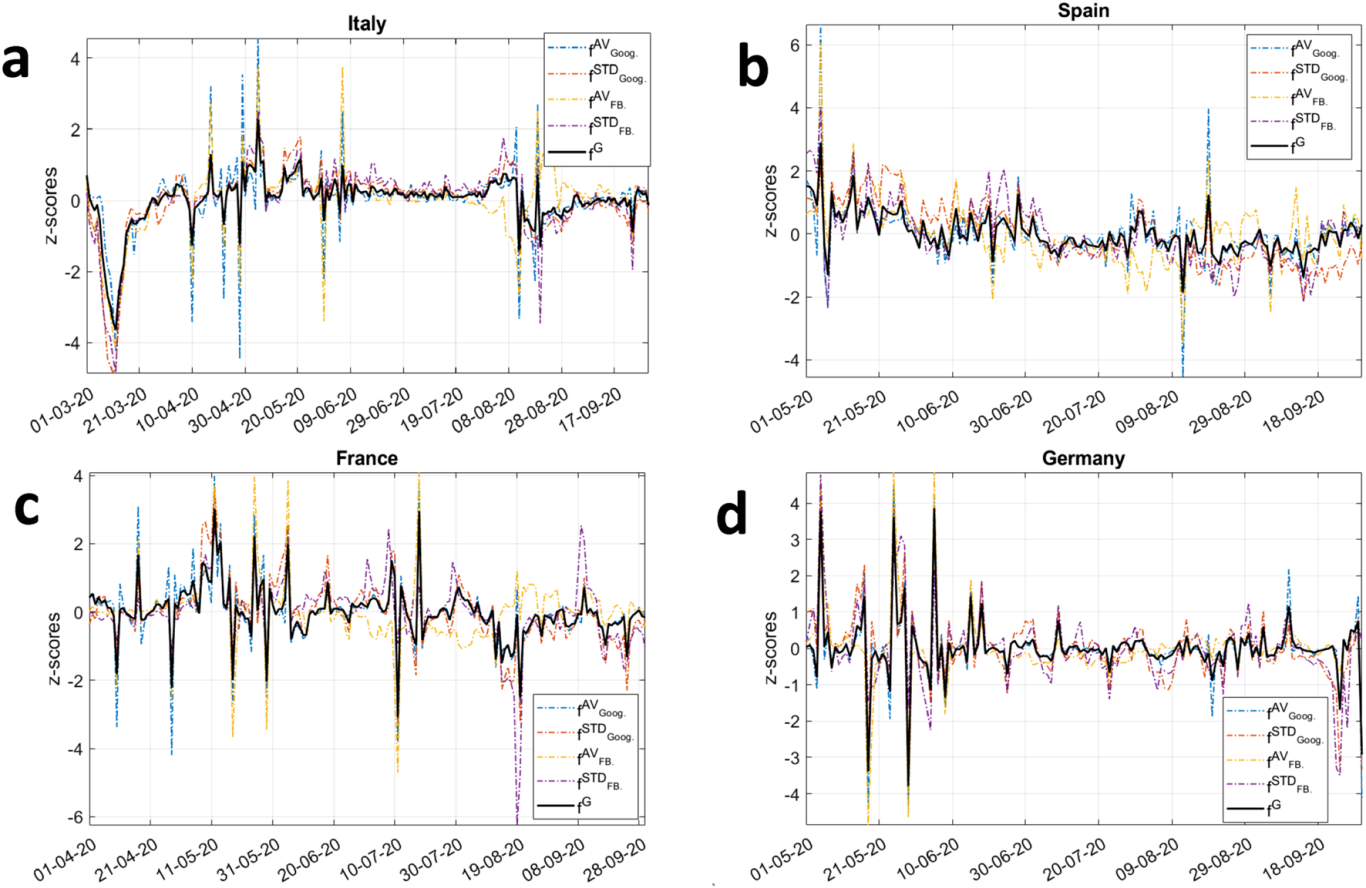
Line diagrams of factor components dynamics. The line diagrams report the in-sample estimates of the global common factor component ($f^{G}$), in black, along with the evolution of the factor components related to the average Google mobility trends ($f^{AV_{GOOG}}$), to the Google mobility trends standard deviation ($f^{STD_{GOOG}}$), to the average Facebook commuting trends ($f^{AV_{FB}}$) and to its standard deviation ($f^{STD_{FB}}$). Moreover, panel (**a**) refers to Italy, (**b**) shows the dynamics related to Spain, (**c**) describes the evolution for France and (**d**) that of Germany. For the sake of comparability, the common factor components are expressed in terms of z-scores.

Before illustrating the daily nowcasts and forecasts of changes in the value of industrial production, we focus on the behavior of the common factor dynamic in the European countries under investigation. This step is instrumental for assessing the day-by-day change $y_t^M$ of the industrial production since the nowcast of $y_t^M$ is the orthogonal projection of such variable on common factors^42^. Figure 6 reports the z-score of the global factors ($f^{G}$) estimated from the dynamic factor model (solid black) using data up to September. The colored dotted lines report the evolution of the factor components related to the average Google mobility trends ($f^{AV_{GOOG}}$), to the Google mobility trends standard deviation ($f^{STD_{GOOG}}$), to the average Facebook commuting trends ($f^{AV_{FB}}$) and to its standard deviation ($f^{STD_{FB}}$), plotted in standard deviations from their mean. The figure shows that, overall, the common factors exhibit high levels of variability during the first and second wave periods, interspersed with a relatively more tranquil dynamics in the summer period. The magnitude of the common factor is quite comparable across the four countries, although its evolution is strongly country-specific. In particular, we observe a significant decrease of the common factor for Italy at the beginning of the sample meaning that, in this particular phase, most of the mobility-related data exhibit large variations leading to its contraction. Moreover, a similar but gentler pattern is also noticeable during the end of August. Spain shows the least fluctuating common factor, especially up to autumn, whereas France displays an oscillating common factor, not only at the dates of the introduction or lifting of mobility restriction measures, but also during the summer phase. As far as Germany, we observe two distinct regimes: a high volatility from the beginning of the sample until the month of June, highlighting a large heterogeneity in human mobility behaviors, and a low volatility afterwards, when new behavioral routines of people became more established.

**Figure 7:**
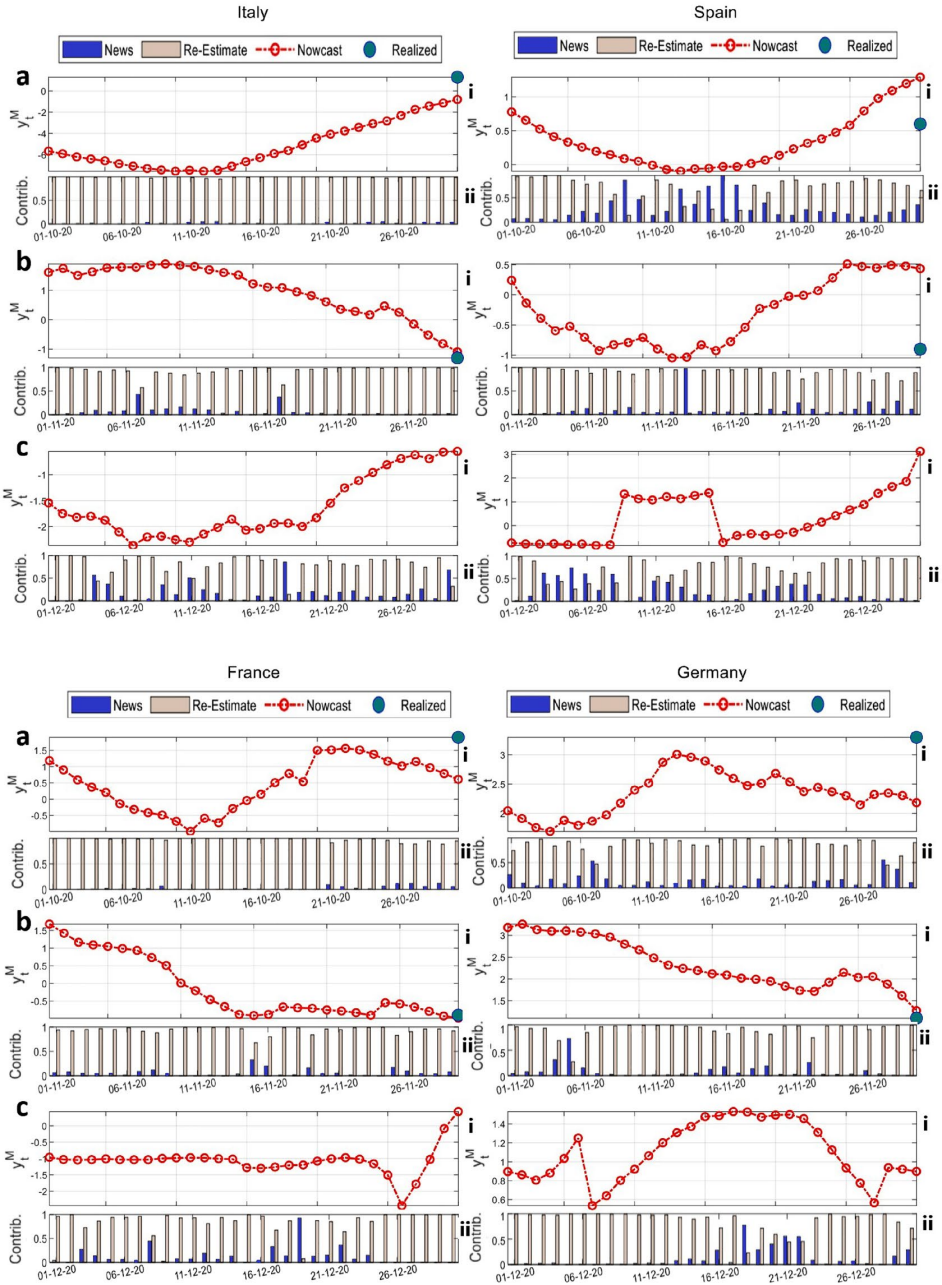
Line diagrams and bar-charts of nowcasting and forecasting results. The line diagrams report the time series of nowcasts and forecasts of the Industrial Production Index changes for Italy, Spain, France and Germany. In particular, panels (**a**) and (**b**) show the nowcasting results for the months of October and November 2020, respectively, while panel (**c**) displays the forecasting results for December 2020, for which no official data is available yet. Red lines in panels (**i**) illustrate the dynamics of the daily nowcasts (and forecasts) for the change in Industrial Production Index ($y^{M}_t$). Colored bars in panels (**ii**) represent the contribution (*Contrib*.) of each component according to Eq. 1 to the daily change in the Industrial Production Index in relative terms. The news effect is reported as a blue bar while the re-estimate contribution is in pink color. Results are obtained by selecting, for each country, the best performing model in terms of mean squared error as per the model selection procedure illustrated in Fig. 8.

In Fig. 7 we present our out-of-sample nowcasting results for countries’ industrial production change in October (Fig. 7a), November (Fig. 7b) and December (Fig. 7c) 2020. Within our framework, the econometric model is re-estimated each day with the input of new data, hence variations of a country’s industrial production result from the combination of news and model re-estimate effects (see Methods). We therefore illustrate with two different bars the contributions of news (blue bar) and model re-estimates (pink bar) to the daily changes in industrial production. The red lines show the evolution of our nowcasts for the daily changes in industrial production for the months of October and November, as well as the forecasts related to the month of December, for which no official figure has been released at the time of writing the present manuscript. Blue dots, instead, represent the realized monthly industrial production percentage change with respect to the previous month.

**Figure 8:**
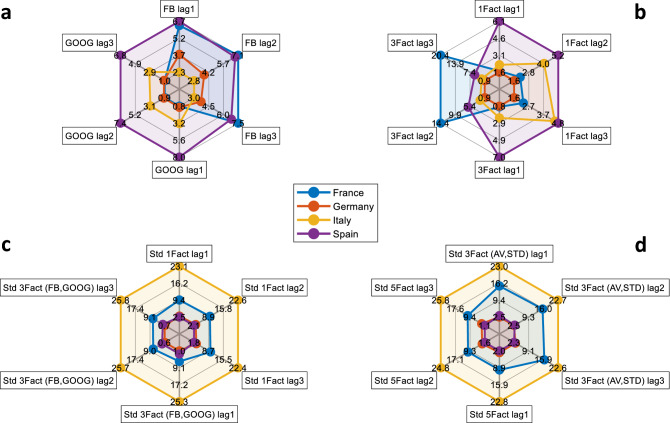
Spider-plots of the model selection results. The figure shows the spider-plots of the values of the mean squared error (MSE) for different types of model configurations, across the four countries. (**a**) shows the MSEs of the models with either Facebook (FB) or Google (GOOG) average mobility data and varying number of lags of the predictors from 1 to 3. (**b**) illustrates the MSEs of the models obtained by including both dataset with either 1 or 3 factors and different number of lags of the predictors from 1 to 3. (**c**) shows the MSEs of the models with either 1 or 3 factors including the time series standard deviations and different number of lags of the predictors from 1 to 3. Finally, (**d**) shows the MSEs of the models with either 3 or 5 factors including the time series standard deviations and different number of lags of the predictors from 1 to 3.

Evidence shows fairly accurate results, as well as that shocks in commuting flows and mobility trends impact the dynamics of the industrial production in an heterogeneous way, depending on the country under consideration, and the time span analyzed. On the one hand, we observe that, overall, Spain seems to be the country mostly affected by mobility shocks, followed by Germany and France. On the other hand, the dynamics of the Italian industrial production is mostly determined by the model re-estimation procedure. In general, news deriving from commuting patterns and mobility flows were particularly relevant to the industrial production in December, following the entangled restrictive measures in view of Christmas. Empirical outcomes also highlight the large intra-month variability of the industrial production dynamics, of which, in general, only the monthly level is known by policymakers, and not without any delay. This highlights the importance of our approach as a higher-frequency indicator of economic activity during pandemic and emergency times, enhancing government’s decision-making processes based on real-time data evidence.

The latter results are obtained by means of the model selection procedure illustrated in Fig. 8. In other words, we investigate the predictive performance of several model specifications and select the variables’ schemes yielding to the best fit in terms of mean squared error (MSE). Particularly, we examine 24 different model configurations by considering either the Facebook mobility data or the Google ones or both, either 1, 3 or 5 common factors, the number of lagged predictors from 1 to 3, the model with or without the corresponding time series standard deviations. In Supplementary Table T3 we report the models ID as indicated in Fig. 8 together with the corresponding explanatory variables inserted into the model and the specification of the number of lags and common factor structures. Evidence shows that results are sensitive to the model specification chosen, thus raising the need to perform a model selection procedure to ensure the best fitting possible. In particular, the best fitting model for France encompasses only Google variables with a common global factor and one lag. For Italy the lowest MSE is obtained by exploiting information of both type of data (FB and GOOG) with three common factors ($f^{G}$, $f^{FB}$ and $f^{GOOG}$) and three lags. Nowcasts and forecast for the German industrial production reported in Fig. 7 are obtained inserting both the averages and the standard deviations of Facebook and Google variables as for Spain.

## Conclusion

A key challenge at the center of political debates in the midst of the pandemic is to which extent health outcomes should be balanced against the economic sacrifices coming with lockdowns and restrictive measures. While some argue that saving lives must take precedence over economic goals, many others insist that priority should be given to preserving the economy. However, due to the unavailability of timely and robust real-time economic data, most policy decisions undertaken by governments so far were solely based on data about the evolution of the epidemics, without knowing the actual magnitude of economic losses such decisions may cause.

Against this background, real human mobility data can be of extreme relevance not only to track the evolution of the epidemics, but also to monitor the contraction of economic activity due to policy restrictions over time. Thus, we introduce a nowcasting framework which leverages a set of real-time human mobility indicators with the aim of timely monitoring economic activity, as measured by the industrial production of a country, on a day-by-day basis. Our approach takes root from the dynamic factor model, a state-of-the-art technique from the statistical domain which allows us to derive an early real-time estimate for the economic activity before the official figure is released.

Our results provide evidence of a large variability of daily economic activity, as well as on country-specific features of economic dynamics, which can be captured by variations in different dimensions of human mobility. This emphasizes the role of our nowcasting method as a timely monitor of economic activity amid the pandemic period, given its ability to quantify the economic downturns and recoveries due to the imposition and lifting of the complex variety of policy restrictions adopted by worldwide governments. The combination of real-time representations of a country’s health and economic conditions given by epidemiological and economic models enable policymakers to promptly tune their decisions, based upon the interplay between health and economic damages generated by their policy decisions.

Despite the merits of our approach to unveil the nexus between mobility variables and industrial production during the pandemic phase, we acknowledge the limitations of the proposed methodology. First, although mobility can be considered a good predictor for industrial production and, therefore, for economic activity, this relationship is informationally stronger with worsening conditions of the pandemic and consequent closures. The potential use of several additional business and economic predictors of the industrial production can tackle the issue of nowcasting beyond hard SARS-CoV-2 phases, although the scarcity of high-frequency economic data raises the need to seek for other variables which might well capture the dynamics of economic indices on a real-time basis. The lack of data is also source of low-granularity of the nowcasting results, as the presence of regional or county-level economic time series data, combined with data on human mobility at an administrative level, would enable a more granular representation of a country’s economic activity.

## Methods

### The nowcasting framework

Our real-time monitoring approach is grounded on the nowcasting methodology, a technique previously employed in meteorology, defining the prediction of the present, the very near future and the very recent past^41^. This approach is relevant when key statistics on the present state of the economy are available with a significant delay and with a low frequency^42^, i.e. at a monthly or quarterly frequency.

In order to estimate the day-by-day impact that mobility variation exerts on industrial production, we exploit the fact that these data series co-move quite strongly with mobility patterns during the pandemic period, so that their behaviour can be captured by few latent factors. In particular, by means of the dynamic factor model^40^, we assume that the information of both mobility patterns and of industrial production - despite being released with different time frequency, i.e. “high” and “low” frequency - can be described by employing a number of latent factors which follow a time series autoregressive process. An important motivation for considering dynamic factor models is that, by knowing the dynamics of latent factors, one can make efficient forecasts for an individual variable through the projections of that variable on the lagged factors. Dynamic factor models have the twin appeals of being grounded in dynamic macroeconomic theory and providing a good first-order description of empirical macroeconomic data, in the sense that a small number of factors explain a large fraction of the variance of many macroeconomic series^49^.

Casting the model in a state space framework allows us to build a formalisation on how market participants read data releases in real time, which involves: monitoring many data, forming expectations about them and revising the assessment on the state of the economy whenever realizations diverge sizeably from those expectations. This is possible because, for a model in a state space representation, the Kalman filter^50^ generates projections for all the variables considered and therefore allows to compute, for each data release, a model-based surprise, i.e. the “news”. Further, nowcast revisions can be expressed as a weighted average of these news^41^. When a value is missing, the Kalman filter replaces the missing value with an estimate and, together with a smoother, one can cope with mixed frequency data series. This feature enables the model to easily handle missing data points and gradually improve the measure of economic activity in a particular month, as new data is released. The model computes a joint forecast of predictors and target series at each release, along with the surprise component of the published data release, which represents the “news” effect. The revision of the nowcast for a low frequency variable can then be described as the product of the weight of each series, estimated on historical data, and the news component at each data release.

Let us now denote $\Psi _v$ the information set at time *v* containing the values assumed by mobility predictors and industrial production up to time *v*. The nowcasting of $y^M_t$, i.e. the daily change of industrial production, consists of the orthogonal projection of $y^M_t$ on $\Psi _v$ given the set of parameter estimates $\theta$^42^. Under the assumption that the data generating process is given by Eq. (8) in Methods, with $\theta$ equal to its quasi-maximum likelihood estimate, the Kalman filter and smoother can be used to obtain in an efficient and automatic manner this projection for any pattern of data available in $\Psi _v$. Another important feature of the nowcasting process is that a sequence of nowcasts is updated as new data are fed into the model. In other words, we perform a sequence of projections $[y_t^M|\Psi _v]$, $[y_t^M|\Psi _{v+1}],\ldots ,$. Nowcast updates are generally influenced by the model’s forecast errors corresponding to each data release and the effects of parameters re-estimation. Suppose at time $v+1$ new data are released, ${x_{j,v+1},j \in {\mathcal {J}}_{v+1}}$, where *j* is the variable for which data are released and ${\mathcal {J}}$ the set of data released, then $\Psi _v \subset \Psi _{v+1}$ and $\Psi _v \setminus \Psi _{v+1} = {x_{j,v+1},j \in {\mathcal {J}}_{v+1}}$. The nowcast update is given by a revision effect plus a parameter re-estimation effect:

1 $$\begin{aligned} \underbrace{(y_t^M|\Psi _{v+1},\theta _{v+1})}_\text {Update Nowcast} = \underbrace{(y_t^M|\Psi _{v},\theta _{v})}_\text {Old Nowcast}+\underbrace{(y_t^M|\Psi _{v},\theta _{v+1})}_\text {Parameters Re-estimation}+\underbrace{\sum _{j \in {\mathcal {J}}_{v+1}} \delta _{j,t,v+1}[x_{j,v+1} - (x_{j,v+1}|\Psi _{v})]}_\text {News Impact} \end{aligned}$$

The nowcast revision is a weighted sum of the news associated with the data release for each variable, while the effect of re-estimation is the difference between the nowcast obtained using the old information set,$\Psi _v$, and the old parameter estimates, $\theta _v$, and the nowcast using the old information set, $\Psi _v$, and the new parameter estimates, $\theta _{v+1}$.

### The dynamic factor model

Dynamic factor models (DFMs) are a class of statistical models for multivariate time series analysis in which the observed endogenous variables are linear functions of some unobserved factors, which have a vector autoregressive structure. The unobserved factors instead are a function of exogenous covariates. Dynamic-factor models can be seen as a dimension reduction technique which is nowadays commonly used by public and private institutions as central banks and investment banks for analysing large panels of time series. In our specific case, industrial production is driven by few factors representing mobility dimensions plus some measurement errors. The premise of a dynamic factor model is that a set of latent dynamic factors drive the co-movements of a high-dimensional vector of time-series variables, which is also affected by a vector of zero-mean idiosyncratic disturbances.

We start by characterizing the dynamics for the daily variables. Let $x_t = (c^{B}_{k,t} , c^{W}_{k,t} , d^{B}_{k,t} , d^{W}_{k,t} , g_{k,t} , r_{k,t} , s_{k,t} ,w_{k,t})'$ denote the vector of (stationary) daily variables of mobility data derived from Facebook and Google, with the indicator $t = \{1,\ldots ,T\}$ being the index variable representing time and $k=(av,std)$, where *av* denotes the sample average and *std* the sample standard deviation. We assume that $x_t$ obeys the following factor model representation:

2 $$\begin{aligned} x_t = \mu + \Gamma f_t + \epsilon _t \end{aligned}$$

where $f_t$ is a $r\times 1$ vector of (unobserved) common factors, $\Gamma$ is a $n\times r$ matrix of factor loadings and $\epsilon _t$ is a vector of idiosyncratic components, and $\mu$ is a vector of unconditional means. Further, the factors *f* are modelled as a Vector Autoregressive (VAR) process of order *p*:

3 $$\begin{aligned} f_t = A_1 f_{t-1} +\cdots + A_p f_{t-p} +u_t \end{aligned}$$

where $A_1,\ldots ,A_p$ are $r\times r$ matrices of autoregressive coefficients and $u_t \sim i.i.d. {\mathcal {N}}(0,Q)$ is an independent and identically distributed term with zero mean and variance *Q*. Also the dynamics of the idiosyncratic component of daily variables $\epsilon _t$ follows an AR(1) process:

4 $$\begin{aligned} \epsilon _{i,t} = \alpha _i \epsilon _{i,t-1} + e_{i,t} \end{aligned}$$

with $(e_{i,t},e_{j,s})=0$, for $i\ne j$ indicating the absence of correlation between the *i*th and the *j*th error components at time *t* and *s*, respectively.

To account for the local cross-sectional correlation within the variables of the two different datasets, which is helpful for a more efficient extraction of the global factor, we further partition $f_t$ into a global factor $f^G$ and mutually independent specific variable factors $f^k$, with $k=\{AV_{FB}, STD_{FB}, AV_{GOOG},STD_{GOOG},AV_{FB},STD_{FB}, AV_{GOOG} ,STD_{GOOG}\}$ (hereafter, we suppress superscripts for sake of readability).

With the aim of including the low frequency economic variables, we express the monthly industrial production variable in terms of its partially-observed daily counterpart^51^. We assume that the unobserved daily growth rate of industrial production $y_t = \Delta Y^D_t$ admits the same factor model representation as the monthly real variables:

5 $$\begin{aligned} \begin{aligned} y_t = \mu _M + \Gamma _M f_t + \epsilon _t^M\\ \epsilon _t^M = \alpha \epsilon _{t-1}^M + e^M_t \end{aligned} \end{aligned}$$

with $$\Gamma _M=\begin{pmatrix} \Gamma _{M,G}&0&0 \end{pmatrix}$$ and $e^M_t \sim i.i.d. {\mathcal {N}}(0,\sigma ^2_M)$. Where the symbol 0 represents a scalar which indicates the absence of dependence between the unobserved daily growth rate of industrial production and mobility specific factors and $e^M_t$ is an independent and identically distributed term with zero mean and variance $\sigma ^2_M$. To link $y_t$ with the observed industrial production we build a partially observed daily time series:

6 $$\begin{aligned} y_t^M = {\left\{ \begin{array}{ll} Y_t^M-Y_{t-30}^M, &{} \text{ if } t=30,60,\ldots \\ {\widetilde{y}}_t^M, &{} \text{ otherwise } \end{array}\right. } \end{aligned}$$

where $Y^M$ is the industrial production in levels and ${\widetilde{y}}_t^M$ represents a missing observation. Here we approximate each month with 30 days length^51^.

7 $$\begin{aligned} y_t^M=y_t+2y_{t-1}+\cdots +29y_{t-28}+30y_{t-29}+29y_{t-30}+\cdots +y_{t-60} \end{aligned}$$

with $t = 30,60,90,\ldots$. For estimating the DFM we cast the Eqs. (2)–(7) in a state space representation. Let ${\hat{x}}=(x_{t'},y_{t}^{M'})$ and ${\hat{\mu }}=(\mu ',\mu _M')$ state space representation results in:

8 $$\begin{aligned} \begin{aligned} {\hat{x}}_t={\hat{\mu }}+Z(\theta )\alpha _t\\ \alpha _t=T(\theta )\alpha _{t-1}+\eta _t \end{aligned} \end{aligned}$$

with $\eta _t \sim i.i.d. {\mathcal {N}}(0,\Sigma _{\eta }^2(\theta ))$ where the vector of states includes the common factors and the idiosyncratic components and all model parameters are collected in $\theta$. The details of the state space representation are provided in SI. We estimate $\theta$ by maximum likelihood implemented by the Expectation Maximisation (EM) algorithm^52–54^. Maximum likelihood allows us to easily deal with such features of the model as substantial fraction of missing data. Given an estimate of $\theta$, the nowcasts as well as the estimates of the factors or of any missing observations in ${\hat{x}}_t$, can be obtained from the Kalman filter or smoother. Details about the model state space representation of the EM algorithm are contained in SI.

### Data description

#### Population commuting patterns

We analyzed data based on the “Coronavirus Disease Prevention Maps” made available by Facebook as a part of its “Data For Good” program, a collection of unique dynamic spatial-temporal datasets illustrating worldwide populations commuting patterns over the COVID-19 pandemic period. The maps use anonymized and aggregated data on mobile-phone-based geo-localized movements of people having their geo-positioning option enabled across country administrative polygons within time intervals of 8 hours, which we aggregate to daily frequency. We collected data relative to commuting patterns in Italy, Spain, France and Germany until 7 January 2021, with different starting points in 2020 depending on the availability of Facebook Coronavirus Disease Prevention Maps (Italy: 24 February; Spain: 12 March; France: 5 March; Germany: 25 March).

#### Human mobility trends

Google has recently disclosed its Community Mobility Reports, an unprecedented phone-tracking based source of mobility data which aggregates anonymized information from users who have turned on their location history setting. In particular, data outline daily changes in mobility of a bunch of geographies across location categories, including retail and recreation, grocery stores and pharmacies, workplaces and transit stations, tracking people’s change in movement trends throughout the pandemic. The baseline value represents the median values on a five-week period from 3 January 2020 to 6 February 2020, constituting a “normal” business period, taking also into account for the different behavior routines on weekdays rather than weekends. We collected data about mobility trends at administrative regional level of Italy, Spain, France and Germany from 15 February 2020 to 7 January 2021.

Both Facebook and Google do not share data with personal identifying information such as a person’s location, contacts or travel. Indeed, their reports are based on aggregated and anonymized datasets of users who have the “Location History” setting turned on.

#### Real economy

We have analyzed as indicator of real economic activity the monthly time series of the Organisation for Economic Co-operation and Development (OECD) Industrial Production Index of Italy, Spain, France and Germany over the period ranging from March to November 2020. Industrial production refers to the output of industrial establishments and covers sectors such as mining, manufacturing, electricity, gas and steam and air-conditioning. It is amongst the most widely employed and monitored macroeconomic indicators, given its ability to represent a country’s economic activity in terms of industrial output, regardless of price changes. The indicator is measured against a reference period (100=2015) that expresses change in the volume of production output with respect to the baseline value.

#### Government policy actions

The Oxford COVID-19 Government Response Tracker (OxCGRT) collects publicly available information on 19 indicators of government responses. Eight of the policy indicators (C1-C8) record information on containment and closure policies, such as school closures and restrictions in movement. Four of the indicators (E1-E4) record economic policies, such as income support to citizens or provision of foreign aid. Seven of the indicators (H1-H7) record health system policies such as the COVID-19 testing regime, emergency investments into healthcare and, most recently, vaccination policies^55^.

## Supplementary information

Supplementary information 1.

## Data Availability

Facebook human mobility data are provided under an academic license agreement with Facebook in the context of the “Facebook Data for Good” program, through which data are released by Facebook upon request to non-profit organizations and academics - see https://dataforgood.fb.com/tools/disease-prevention-maps/. Google human mobility data are publicly available through the “Google Mobility Reports” program at https://www.google.com/covid19/mobility/. Policy data are publicly available and provided by the University of Oxford in co-operation with the Blavatnik School of Government through the “Oxford COVID-19 Government Response Tracker” at https://www.bsg.ox.ac.uk/research/research-projects/covid-19-government-response-tracker. Real economy data are publicly available and provided by the OECD at https://stats.oecd.org/.
