# Supplementary material for: Mobility-based real-time economic monitoring amid the COVID-19 pandemic
Source: Sci Rep. 2021 Jun 22;11:13069. doi: 10.1038/s41598-021-92134-x (PMC8219782; doi:10.1038/s41598-021-92134-x)
Supplement: Supplementary file 1 — Supplementary information 1. [file 41598_2021_92134_MOESM1_ESM.pdf]

Supplementary information
Mobility-based real-time economic monitoring amid the COVID-19 pandemic

Spelta A., Pagnottoni, P.

1 Supplementary Information

State space representation of the model

Below are the details for the state space representation of equation (8) as specified by the Eqs. (2)-(7), for $p = 1$, $r = 3$ and a single monthly variable y_t^M. This means that the following formulation refers to one lag common factors, which collect the global component f^G and discriminate between FB and Google variables f^{FB} and f^{GOOG}. The state space representation of equation (8) articulates as follows:

$$\underbrace{\begin{pmatrix} x_t \\ y_t^M \end{pmatrix}}_{\hat{x}_t} = \underbrace{\begin{pmatrix} \mu \\ \mu_M \end{pmatrix}}_{\hat{\mu}} + \underbrace{\begin{pmatrix} \Gamma & \cdots & 0 & \cdots & 0 & I_n & 0 & \cdots & 0 & \cdots & 0 \\ \Gamma_M & \cdots & 30\Gamma_M & \cdots & \Gamma_M & 0 & 1 & \cdots & 30 & \cdots & 1 \end{pmatrix}}_{Z(\theta)} \underbrace{\begin{pmatrix} f_t \\ \vdots \\ f_{t-31} \\ \epsilon_t \\ \epsilon_t^M \\ \vdots \\ \epsilon_{t-31}^M \end{pmatrix}}_{\alpha_t} \tag{1}$$

$$\begin{pmatrix} f_t \\ \vdots \\ f_{t-31} \\ \epsilon_t \\ \epsilon_t^M \\ \vdots \\ \epsilon_{t-31}^M \end{pmatrix} = \underbrace{\begin{pmatrix} A_1 & 0 & 0 & 0 \\ diag(I_r) & 0 & 0 & 0 \\ 0 & diag(\alpha_1), ..., diag(\alpha_n) & 0 & 0 \\ 0 & 0 & \alpha_M & 0 \\ 0 & 0 & I_D & 0 \end{pmatrix}}_{T(\theta)} \begin{pmatrix} f_{t-1} \\ \vdots \\ f_{t-32} \\ \epsilon_{t-1} \\ \epsilon_{t-1}^M \\ \vdots \\ \epsilon_{t-32}^M \end{pmatrix} + \underbrace{\begin{pmatrix} u_t \\ 0 \\ \vdots \\ e_t \\ e_t^M \\ \vdots \\ 0 \end{pmatrix}}_{\eta_t} \tag{2}$$

where $\epsilon = (\epsilon_{1,t}, ..., \epsilon_{n,t})'$ and $e = (e_{1,t}, ..., e_{n,t})'$.

The block specific factor structure further implies that:

$$\Gamma = \begin{pmatrix} \Gamma_{FB,G} & \Gamma_{FB,FB} & 0 \\ \Gamma_{GOOG,G} & 0 & \Gamma_{GOOG,GOOG} \end{pmatrix} \tag{3}$$

$$\Gamma_M = \begin{pmatrix} \Gamma_{M,G} & 0 & 0 \end{pmatrix} \tag{4}$$

$$f_t = \begin{pmatrix} f_t^G \\ f_t^{FB} \\ f_t \end{pmatrix} \tag{5}$$

$$A_1 = \begin{pmatrix} A_{1,G} & 0 & 0 \\ 0 & A_{1,FB} & 0 \\ 0 & 0 & A_{1,GOOG} \end{pmatrix} \tag{6}$$

$$Q = \begin{pmatrix} Q_G & 0 & 0 \\ 0 & Q_{FB} & 0 \\ 0 & 0 & Q_{GOOG} \end{pmatrix} \tag{7}$$

Expectation Maximization algorithm

The parameters θ of the state space form of equation (8) are estimated by the Expectation Maximisation (EM) algorithm[1, 2, 3]. The algorithm is a popular solution to problems, for which latent or missing data yield a direct maximisation of the likelihood function intractable or computationally difficult. The basic principle behind the EM is to write the likelihood in terms of observable as well as latent variables and given the available data Ψ_v obtain the maximum likelihood estimates in a sequence of two alternating steps. Precisely, iteration $\tau+1$, with $\tau = 1, ..., T$, would consist of the following steps:

- The E-Step in which the expectation of the log-likelihood conditional on the data is calculated using the estimates from the previous iteration $\theta(\tau)$
- The M-Step in which the new parameters, $\theta(\tau+1)$, are estimated through the maximisation of the expected log-likelihood (from the previous iteration) with respect to θ.

We first estimate μ and μ_M by sample means and use the de-meaned data throughout the EM steps. To deal with missing observations in $\hat{x}_t$ we introduce selection matrices[4] W_t and W_t^M. They are diagonal matrices of size n and 1, respectively, with ones corresponding to the non-missing values in x_t and y_t^M, respectively. For the sake of simplicity, we first consider the case without restrictions on Γ, Γ_M, A_1 and Q implied by block specific factors. To account for the restrictions imposed by group specific factors, we need to split the parameters in Γ into blocks and repeating the computations for each block. The matrix of loadings for the daily variables assumes the following form:

$$vec(\Gamma(\tau+1)) = \left(\sum_{t=1}^{T} {}_{\theta(\tau)}[f_t f_t'|\Psi_v] \otimes W_t\right)^{-1} vec\left(\sum_{t=1}^{T} W_t x_{t\theta(\tau)}[f_t'|\Psi_v] + W_{t\theta(\tau)}[\epsilon_t f_t'|\Psi_v]\right) \tag{8}$$

For deriving the matrix of loadings for the monthly variables, let $f_t^p = [f_t', ..., f_{t-p+1}']'$ and $D = \sum_{t=1}^{T} {}_{\theta(\tau)}[f_t^{32} f_t^{32\prime}|\Psi_v] W_t^M$ The unrestricted $\hat{\Gamma}_M^{ur} = (\Gamma_M^{ur}, ..., 30\Gamma_M^{ur}, ..., \Gamma_M^{ur})$ is given by:

$$vec(\hat{\Gamma}_M^{ur}(\tau+1)) = D^{-1}\left(\sum_{t=1}^{T} W_t^M y_t^M{}_{\theta(\tau)}[f_t^{32\prime}|\Psi_v]\right) \tag{9}$$

The restricted $\hat{\Gamma}_M$ is given by:

$$\hat{\Gamma}_M(\tau+1) = \Gamma_M^{ur}(\tau+1) - D^{-1}C(CDC')^{-1}C\hat{\Gamma}_M^{ur} \tag{10}$$

where

$$C = \begin{bmatrix} I_r & -I_r/2 & 0 & 0 & 0 & 0 \\ \vdots & 0 & \ddots & 0 & 0 & 0 \\ \vdots & 0 & 0 & -I_r/30 & 0 & 0 \\ \vdots & 0 & 0 & 0 & \ddots & 0 \\ I_r & 0 & 0 & 0 & 0 & -I_r \end{bmatrix} \tag{11}$$

The autoregressive coefficients in the factor VAR are:

$$A_1(\tau+1) = \left(\sum_{t=1}^{T} {}_{\theta(\tau)}[f_t f_{t-1}'|\Psi_v]\right)\left(\sum_{t=1}^{T} {}_{\theta(\tau)}[f_{t-1} f_{t-1}'|\Psi_v]\right)^{-1} \tag{12}$$

The covariance matrix in the factor VAR has the following form:

$$Q(\tau+1) = \frac{1}{T}\left(\sum_{t=1}^{T} {}_{\theta(\tau)}[f_t f_t'|\Psi_v] - A_1(\tau+1)\sum_{t=1}^{T} {}_{\theta(\tau)}[f_{t-1} f_t'|\Psi_v]\right) \tag{13}$$

The autoregressive coefficients in the AR representation for the idiosyncratic component of the daily variables can be written as:

$$\alpha_i(\tau+1) = \left(\sum_{t=1}^{T} {}_{\theta(\tau)}[\epsilon_{i,t}\epsilon_{i,t-1}'|\Psi_v]\right)\left(\sum_{t=1}^{T} {}_{\theta(\tau)}[\epsilon_{i,t}^2|\Psi_v]\right)^{-1} \tag{14}$$

Hence, the variance in the AR representation for the idiosyncratic component of the daily variables is:

$$\sigma_i^2(\tau+1) = \frac{1}{T}\left(\sum_{t=1}^{T} {}_{\theta(\tau)}[\epsilon_{i,t}^2|\Psi_v] - a_i(\tau+1)\sum_{t=1}^{T} {}_{\theta(\tau)}[\epsilon_{i,t-1}\epsilon'_{i,t}|\Psi_v]\right) \tag{15}$$

where $i = 1, ..., n, M$. The conditional expectations (the E-step) in the expressions above are computed using the Kalman smoother on the state space representation of equation (8) with the previous iteration parameters $\theta(\tau)$. The initial parameters $\theta(0)$ are obtained on the basis of principal components analysis[5].

Supplementary Figures and Tables

Variable	Definition
c^B	Number of commuters between (B) administrative regions, Facebook (FB) data
c^W	Number of commuters within (W) administrative regions, Facebook (FB) data
d^B	Distance travelled by commuters between (B) administrative regions, Facebook (FB) data
d^W	Distance travelled by commuters within (W) administrative regions, Facebook (FB) data
g	Mobility trends to groceries and pharmacies (g), Google ($GOOG$) Mobility Reports data
r	Mobility trends to retail stores (g), Google ($GOOG$) Mobility Reports data
s	Mobility trends to transit stations (s), Google ($GOOG$) Mobility Reports data
w	Mobility trends to workplaces (w), Google ($GOOG$) Mobility Reports data
Y	OECD Industrial Production Index in levels, baseline 2015=100
y	OECD Industrial Production Index in expressed as the difference with respect to the previous period
av, std	Denote the 7-day moving average (av), the standard deviation across administrative regions (std)
M,D,t	Denote monthly frequency (M), daily frequency (D), and the measurement time (t)

Supplementary Table 1: **List of variables.** The table shows the list of analyzed variables which are included in the nowcasting model specifications, along with the characterizing subscripts and superscripts.

Country	Jan-2020	Feb-2020	Mar-2020	Apr-2020	May-2020	Jun-2020	Jul-2020	Aug-2020	Sep-2020	Oct-2020	Nov-2020
France	101.7	102.8	85.4	67.7	81.2	91.7	95.1	96.1	97.6	99.5	98.6
Germany	100.9	101.3	90.2	71.5	78.6	87.4	90.1	90.2	92.3	95.6	96.7
Italy	104.7	103.4	74.5	59.6	84.3	91.3	98.2	105.2	99.8	101.1	99.8
Spain	104	103.9	90.3	70.6	80.7	91.7	100.8	101.3	102.2	102.8	101.9

Supplementary Table 2: **Industrial Production Index.** The table shows the dynamics of the OECD Industrial Production Index of France, Germany, Italy and Spain over the period January 2020 - November 2020.

Country	France	Germany	Italy	Spain
France	1			
Germany	0.9470	1		
Italy	0.9529	0.8222	1	
Spain	0.9823	0.9336	0.9299	1

Supplementary Table 3: **Industrial Production Index correlation matrix.** The table shows the pairwise correlations of the OECD Industrial Production Index (IPI) of France, Germany, Italy and Spain over the period January 2020 - November 2020.

Variable	c_{av}^B	c_{av}^W	d_{av}^B	d_{av}^W	g_{av}	r_{av}	s_{av}	w^{av}
Level	0.5150	0.7335	0.6011	0.5222	0.0096	0.7624	0.2671	0.4382
First difference	<0.001	<0.001	<0.001	<0.001	<0.001	<0.001	<0.001	<0.001

Supplementary Table 4: **Stationarity tests.** The table shows the p-values of the Augmented Dickey-Fuller (ADF) unit root tests conducted on the mobility predictor time series expressed in levels and in first differences. Under the null hypothesis, a unit root is present in the time series sample, whereas the alternative hypothesis is stationarity of the considered time series. The model specification includes a constant and no time trend.

**Model ID**	**Variables**	**Lags**	**Factors**
GOOG lag1	r_{av}, g_{av}, w_{av}, s_{av}	1	f^G
FB lag1	c^B_{av}, c^W_{av}, d^B_{av}, d^W_{av}	1	f^G
GOOG lag2	r_{av}, g_{av}, w_{av}, s_{av}	2	f^G
FB lag2	c^B_{av}, c^W_{av}, d^B_{av}, d^W_{av}	2	f^G
GOOG lag3	r_{av}, g_{av}, w_{av}, s_{av}	3	f^G
FB lag3	c^B_{av}, c^W_{av}, d^B_{av}, d^W_{av}	3	f^G
1Fact lag1	r_{av}, g_{av}, w_{av}, s_{av},<br>c^B_{av}, c^W_{av}, d^B_{av}, d^W_{av}	1	f^G
1Fact lag2	r_{av}, g_{av}, w_{av}, s_{av},<br>c^B_{av}, c^W_{av}, d^B_{av}, d^W_{av}	2	f^G
1Fact lag3	r_{av}, g_{av}, w_{av}, s_{av},<br>c^B_{av}, c^W_{av}, d^B_{av}, d^W_{av}	3	f^G
3Fact lag1	r_{av}, g_{av}, w_{av}, s_{av},<br>c^B_{av}, c^W_{av}, d^B_{av}, d^W_{av}	1	f^G, f^{GOOG}, f^{FB}
3Fact lag2	r_{av}, g_{av}, w_{av}, s_{av},<br>c^B_{av}, c^W_{av}, d^B_{av}, d^W_{av}	2	f^G, f, f^{FB}
3Fact lag3	r_{av}, g_{av}, w_{av}, s_{av},<br>c^B_{av}, c^W_{av}, d^B_{av}, d^W_{av}	3	f^G, f^{GOOG}, f^{FB}
Std 1Fact lag1	r_{av}, g_{av}, w_{av}, s_{av},<br>c^B_{av}, c^W_{av}, d^B_{av}, d^W_{av},<br>r_{std}, g_{std}, w_{std}, s_{std},<br>c^B_{std}, c^W_{std}, d^B_{std}, d^W_{std}	1	f^G
Std 1Fact lag2	r_{av}, g_{av}, w_{av}, s_{av},<br>c^B_{av}, c^W_{av}, d^B_{av}, d^W_{av},<br>r_{std}, g_{std}, w_{std}, s_{std},<br>c^B_{std}, c^W_{std}, d^B_{std}, d^W_{std}	2	f^G
Std 1Fact lag3	r_{av}, g_{av}, w_{av}, s_{av},<br>c^B_{av}, c^W_{av}, d^B_{av}, d^W_{av},<br>r_{std}, g_{std}, w_{std}, s_{std},<br>c^B_{std}, c^W_{std}, d^B_{std}, d^W_{std}	3	f^G
Std 3Fact (FB,GOOG) lag1	r_{av}, g_{av}, w_{av}, s_{av},<br>c^B_{av}, c^W_{av}, d^B_{av}, d^W_{av},<br>r_{std}, g_{std}, w_{std}, s_{std},<br>c^B_{std}, c^W_{std}, d^B_{std}, d^W_{std}	1	f^G, f^{GOOG}, f^{FB}
Std 3Fact (FB,GOOG) lag2	r_{av}, g_{av}, w_{av}, s_{av},<br>c^B_{av}, c^W_{av}, d^B_{av}, d^W_{av},<br>r_{std}, g_{std}, w_{std}, s_{std},<br>c^B_{std}, c^W_{std}, d^B_{std}, d^W_{std}	2	f^G, f^{GOOG}, f^{FB}
Std 3Fact (FB,GOOG) lag3	r_{av}, g_{av}, w_{av}, s_{av},<br>c^B_{av}, c^W_{av}, d^B_{av}, d^W_{av},<br>r_{std}, g_{std}, w_{std}, s_{std},<br>c^B_{std}, c^W_{std}, d^B_{std}, d^W_{std}	3	f^G, f^{GOOG}, f^{FB}
Std 3Fact (AV,STD) lag1	r_{av}, g_{av}, w_{av}, s_{av},<br>c^B_{av}, c^W_{av}, d^B_{av}, d^W_{av},<br>r_{std}, g_{std}, w_{std}, s_{std},<br>c^B_{std}, c^W_{std}, d^B_{std}, d^W_{std}	1	f^G, f^{AV}, f^{STD}
Std 3Fact (AV,STD) lag2	r_{av}, g_{av}, w_{av}, s_{av},<br>c^B_{av}, c^W_{av}, d^B_{av}, d^W_{av},<br>r_{std}, g_{std}, w_{std}, s_{std},<br>c^B_{std}, c^W_{std}, d^B_{std}, d^W_{std}	2	f^G, f^{AV}, f^{STD}
Std 3Fact (AV,STD) lag3	r_{av}, g_{av}, w_{av}, s_{av},<br>c^B_{av}, c^W_{av}, d^B_{av}, d^W_{av},<br>r_{std}, g_{std}, w_{std}, s_{std},<br>c^B_{std}, c^W_{std}, d^B_{std}, d^W_{std}	3	f^G, f^{AV}, f^{STD}
Std 5Fact lag1	r_{av}, g_{av}, w_{av}, s_{av},<br>c^B_{av}, c^W_{av}, d^B_{av}, d^W_{av},<br>r_{std}, g_{std}, w_{std}, s_{std},<br>c^B_{std}, c^W_{std}, d^B_{std}, d^W_{std}	1	f^G, $f^{AV_{GOOG}}$, $f^{STD_{GOOG}}$, $f^{AV_{FB}}$, $f^{STD_{FB}}$
Std 5Fact lag2	r_{av}, g_{av}, w_{av}, s_{av},<br>c^B_{av}, c^W_{av}, d^B_{av}, d^W_{av},<br>r_{std}, g_{std}, w_{std}, s_{std},<br>c^B_{std}, c^W_{std}, d^B_{std}, d^W_{std}	2	f^G, $f^{AV_{GOOG}}$, $f^{STD_{GOOG}}$, $f^{AV_{FB}}$, $f^{STD_{FB}}$
Std 5Fact lag3	r_{av}, g_{av}, w_{av}, s_{av},<br>c^B_{av}, c^W_{av}, d^B_{av}, d^W_{av},<br>r_{std}, g_{std}, w_{std}, s_{std},<br>c^B_{std}, c^W_{std}, d^B_{std}, d^W_{std}	3	f^G, $f^{AV_{GOOG}}$, $f^{STD_{GOOG}}$, $f^{AV_{FB}}$, $f^{STD_{FB}}$

Supplementary Table 5: **Model configurations.** The table shows the set of different models tested. The first column refers to the model ID as reported in Fig. 9 of the main text. Columns two, three and four show the variables inserted in each model, the number of lags for the autoregressive components of the common factors, and the number and type of common factors included in the model specification, respectively.

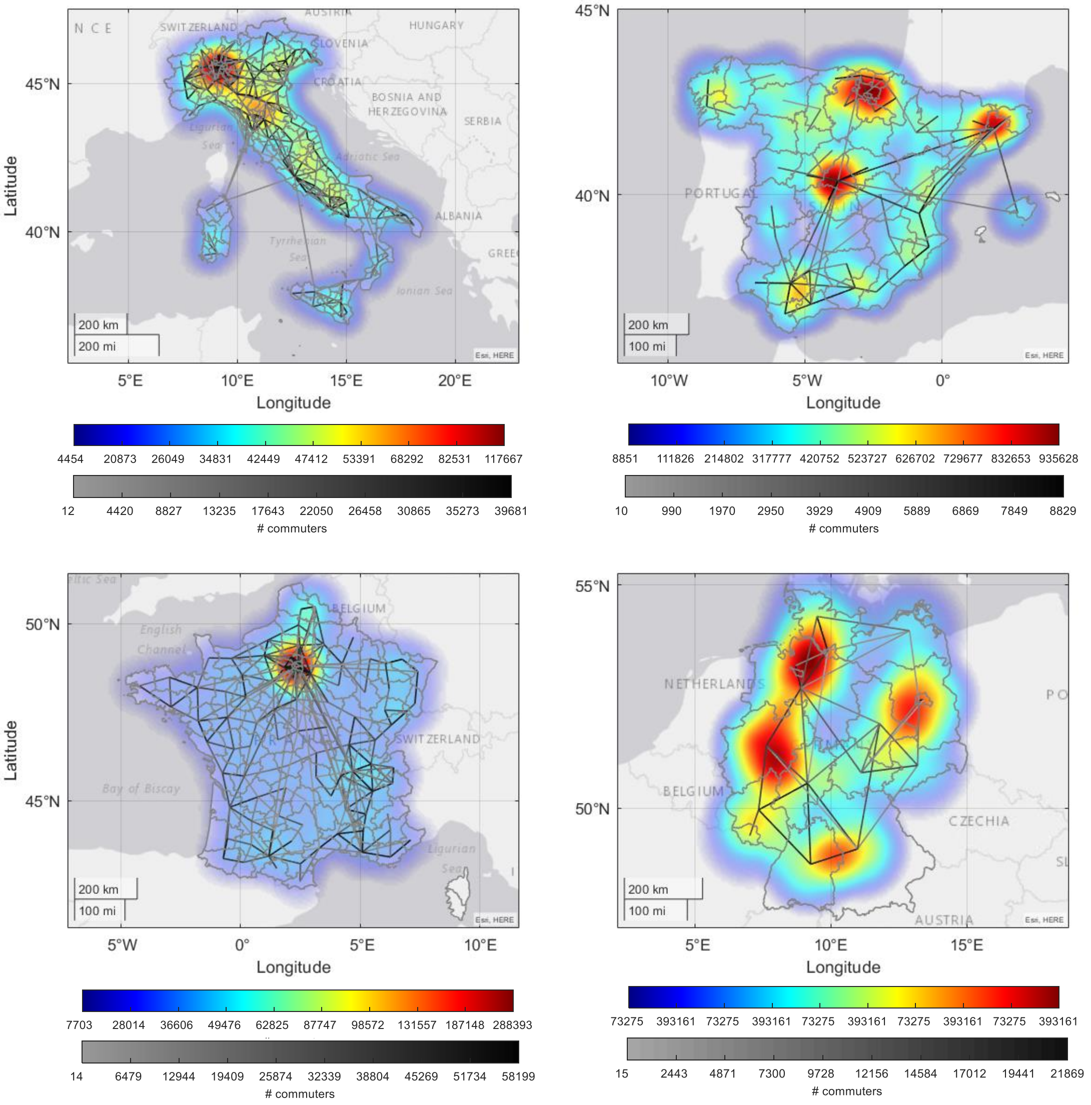

Supplementary Figure 1: **The geography of commuting patterns before the SARS-CoV-2 outbreak.** The figure reports the heatmap and line diagram showing the mobile-phone-based commuting patterns in Italy, Spain, France and Germany during the pre-crisis period, where baselines are derived from 5-to-13 weeks of pre-crisis data. It reports the geographic density plot of commuters within each administrative region, together with the amount of individuals flowing between administrative regions indicated by the colored links.

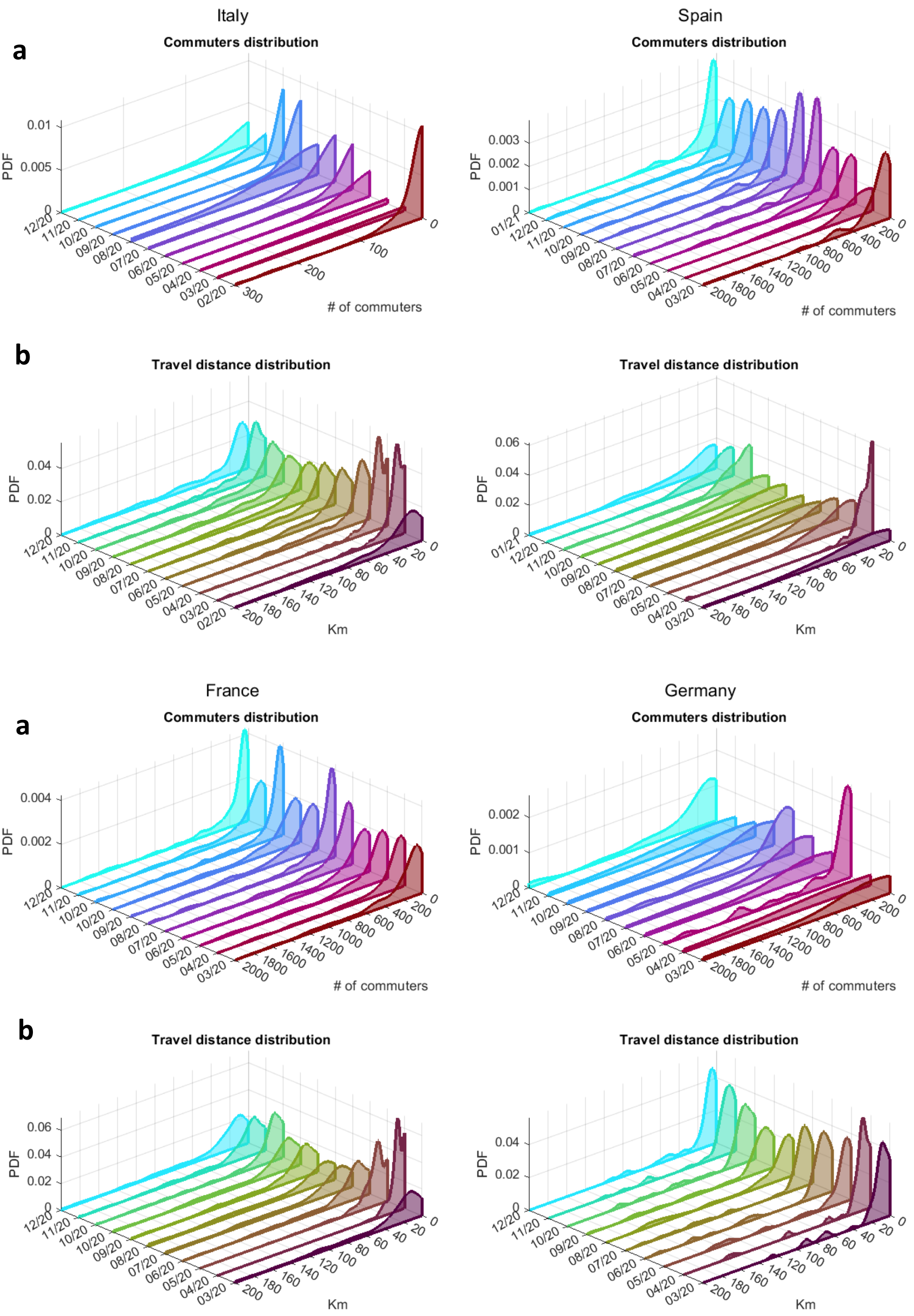

Supplementary Figure 2: **Distribution of mobility patterns over time.** The figure shows the empirical probability distribution function of mobility patterns over the months of the pandemic for Italy, Spain, France and Germany. Panel (**a**) shows the distribution of the overall number of commuters, while Panel (**b**) illustrates the distribution of the overall travel distance.

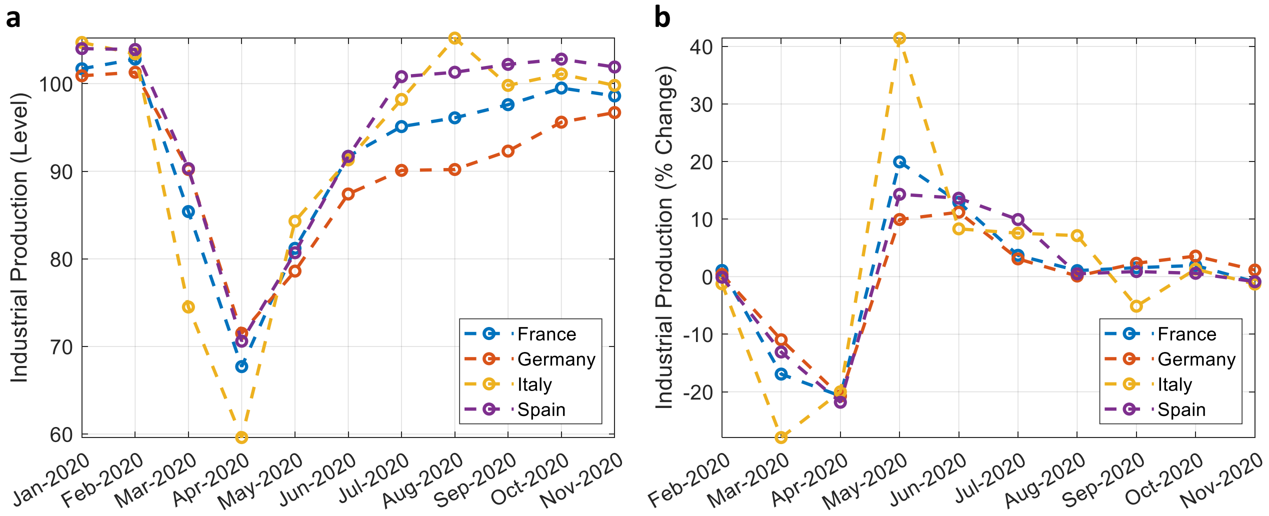

Supplementary Figure 3: **Industrial production dynamics.** The figure shows the line diagram of the time evolution of the OECD Industrial Production Index y_t^M (baseline 100=2015) of France, Germany, Italy and Spain over the period January 2020 - November 2020 in levels (**b**) and in percentage change terms (**b**).

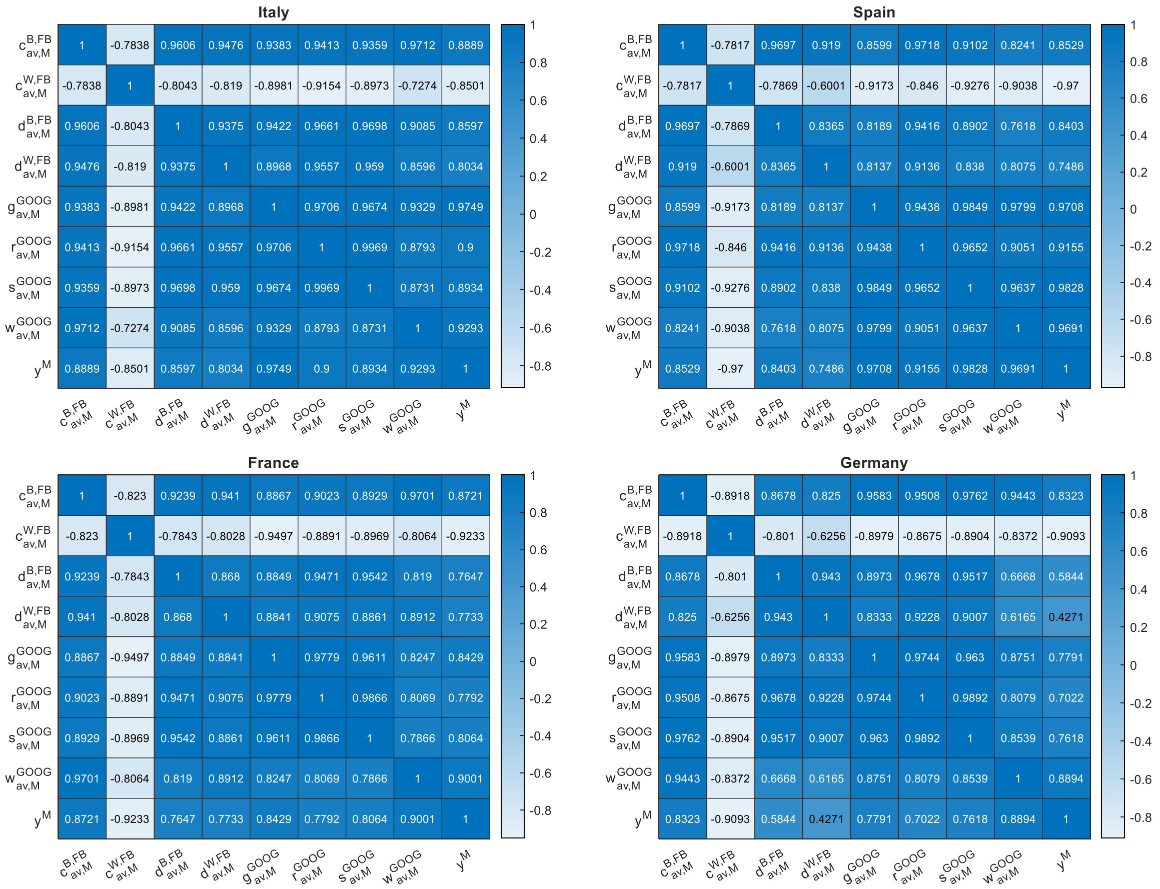

Supplementary Figure 4: **Mobility and Industrial production correlation.** The figure shows the pairwise correlation matrices between the mobility predictors and the OECD Industrial Production Index (y_t^M) in level (baseline 100=2015) over the analyzed sample period.

2 Data representativity

In this section we investigate whether the variables we employed in th work, which are based on phone-tracking movements, are representative and constitute a good proxy for workers travelling and thus commuting on the transport infrastructure; or whether these variables embed information concerning the population densities in different geographical areas.

We study the representativity of Facebook mobility data as a proxy of commuting, by performing a comparison exercises with the 2011 commuting network provided by the Italian National Institute of Statistics (ISTAT), which encodes movements of workers travelling between municipalities, recorded in the last census. We tread carefully in the comparison, first because the commuting network contains information only about residents who travel for working reasons, and second because the information refers to the Italian mobility patterns of 9 years ago. On the other side, this data is not biased towards individuals who own a phone and are registered on social networks, as in Facebook data, therefore we believe it is useful to further validate the representativity of the mobility data observed before entering the lockdown phase. We conduct this further analysis on the Italian mobility network, since it is the only data to us available at present. To make a consistent comparison we filtered out those paths where the number of commuters is less than 10 (indeed Facebook employs this value as threshold to include an observation, see [6]) and retained only municipalities which are present also in our dataset (approx. 1/3 of all Italian municipalities). Besides, we build an averaged graph of mobility over a window of 14 days before the Italian national lockdown of the 10th of March, and we aggregate the network at NUTS3 level. Then, we performed Pearson's correlation test to the following metrics: In-Strength and Out-Strength of nodes, i.e. the number of incoming commuters and outgoing commuters. In all cases we find a significant positive correlation, i.e. 0.74 and 0.62 for In- and Out-Strength respectively, as reported in the left and central panels of Fig. 5.

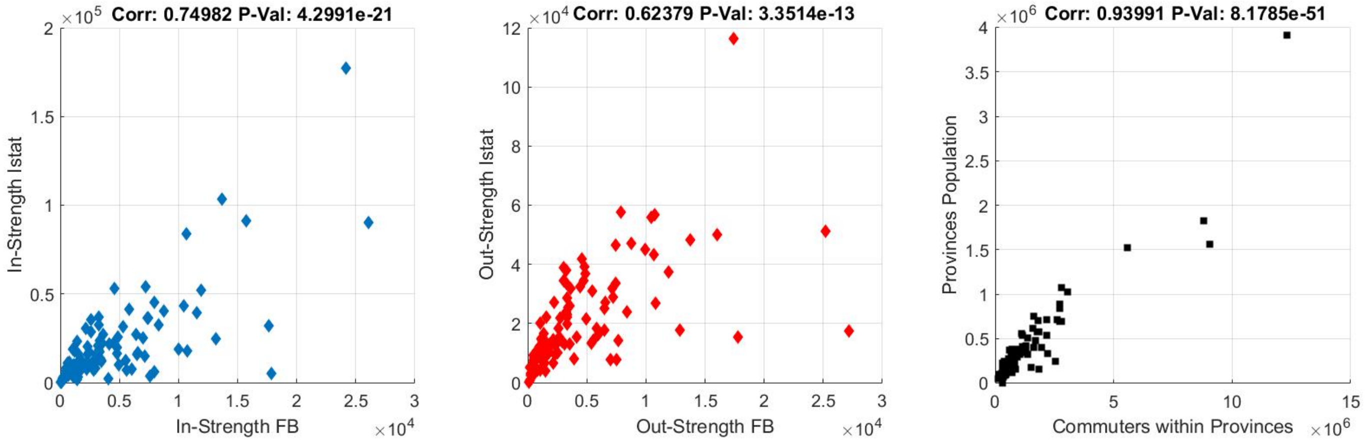

Supplementary Figure 5: **Scatterplot of nodes' In- and Out-Strength in Facebook mobility network before lockdown with respect to the ISTAT commuting network and Facebook commuting within provinces with respect to population.** The figure reports the scatterplots of nodes' In-Strength and Out-Strength of nodes, i.e. the number of incoming commuters and outgoing commuters computed on the Facebook mobility network against the same metrics computed on the ISTAT network. In particular the left panel reports the dispersion between nodes' In-Strength computed on the Facebook (x-axis) commuting network and on the ISTAT network (y-axis). The central panel provides information on the correlation among nodes' Out-Strenght, while the right panel shows the scatterplot of the Facebook commuters within provinces and province population.

Secondly, we further investigate whether commuting patterns observed within provinces in the Facebook

dataset are consistent with the provinces' population. This last information has been obtained from the 15th General Census of Population and Housing developed by the Italian National Institute of Statistics (ISTAT). The database contains information, at sub-municipal levels, on the demographic and social structure of the Italian population. In particular for each district, we rely on information on population size, available in the Employment Register created in 2011 in the occasion of the CIS2011 Virtual Business Census and updated annually, starting from 2012 (see [7]) and we aggregate this information at province (NUTS3) level to be consistent with Facebook data. The right panel of Fig. 5 shows a positive and significant correlation between the two measures (0.93). This validates the use of commuting patterns as key features to provide us with a solid background for our nowcasting model.

Supplementary References

[1] Arthur P Dempster, Nan M Laird, and Donald B Rubin. Maximum likelihood from incomplete data via the em algorithm. *Journal of the Royal Statistical Society: Series B (Methodological)*, 39(1):1–22, 1977.

[2] Robert H Shumway and David S Stoffer. An approach to time series smoothing and forecasting using the em algorithm. *Journal of time series analysis*, 3(4):253–264, 1982.

[3] Mark W Watson and Robert F Engle. Alternative algorithms for the estimation of dynamic factor, mimic and varying coefficient regression models. *Journal of Econometrics*, 23(3):385–400, 1983.

[4] Marta Banbura, D Giannone, and L Reichlin. Nowcasting with daily data. *European Central Bank, Working Paper*, 2011.

[5] Catherine Doz, Domenico Giannone, and Lucrezia Reichlin. A two-step estimator for large approximate dynamic factor models based on kalman filtering. *Journal of Econometrics*, 164(1):188–205, 2011.

[6] Paige Maas, Shankar Iyer, Andreas Gros, Wonhee Park, Laura McGorman, Chaya Nayak, and P Alex Dow. Facebook disaster maps: Aggregate insights for crisis response and recovery. In *Proceedings of the 16th International Conference on Information Systems for Crisis Response and Management (ISCRAM), Valencia, Spain. 2019*, 2019.

[7] Giovanni Bonaccorsi, Francesco Pierri, Matteo Cinelli, Andrea Flori, Alessandro Galeazzi, Francesco Porcelli, Ana Lucia Schmidt, Carlo Michele Valensise, Antonio Scala, Walter Quattrociocchi, et al. Economic and social consequences of human mobility restrictions under COVID-19. *Proceedings of the National Academy of Sciences*, 117(27):15530–15535, Apr 2020.
